# Transmission of *Turnip yellows virus* by *Myzus persicae* Is Reduced by Feeding Aphids on Double-Stranded RNA Targeting the Ephrin Receptor Protein

**DOI:** 10.3389/fmicb.2018.00457

**Published:** 2018-03-13

**Authors:** Michaël Mulot, Baptiste Monsion, Sylvaine Boissinot, Maryam Rastegar, Sophie Meyer, Nicole Bochet, Véronique Brault

**Affiliations:** ^1^SVQV, Université de Strasbourg, Institut National de la Recherche Agronomique, Colmar, France; ^2^Department of Plant Protection, Shiraz University, Shiraz, Iran

**Keywords:** polerovirus, virus transmission, virus receptor, RNA interference, transmission inhibition, plant viruses, aphid vector

## Abstract

Aphid-transmitted plant viruses are a threat for major crops causing massive economic loss worldwide. Members in the *Luteoviridae* family are transmitted by aphids in a circulative and non-replicative mode. Virions are acquired by aphids when ingesting sap from infected plants and are transported through the gut and the accessory salivary gland (ASG) cells by a transcytosis mechanism relying on virus-specific receptors largely unknown. Once released into the salivary canal, virions are inoculated to plants, together with saliva, during a subsequent feeding. In this paper, we bring *in vivo* evidence that the membrane-bound Ephrin receptor (Eph) is a novel aphid protein involved in the transmission of the *Turnip yellows virus* (TuYV, *Polerovirus* genus, *Luteoviridae* family) by *Myzus persicae*. The minor capsid protein of TuYV, essential for aphid transmission, was able to bind the external domain of Eph in yeast. Feeding *M. persicae* on *in planta*- or *in vitro*-synthesized dsRNA targeting *Eph*-mRNA (dsRNA_Eph_) did not affect aphid feeding behavior but reduced accumulation of TuYV genomes in the aphid's body. Consequently, TuYV transmission efficiency by the dsRNA_Eph_-treated aphids was reproducibly inhibited and we brought evidence that Eph is likely involved in intestinal uptake of the virion. The inhibition of virus uptake after dsRNA_Eph_ acquisition was also observed for two other poleroviruses transmitted by *M. persicae*, suggesting a broader role of Eph in polerovirus transmission. Finally, dsRNA_Eph_ acquisition by aphids did not affect nymph production. These results pave the way toward an ecologically safe alternative of insecticide treatments that are used to lower aphid populations and reduce polerovirus damages.

## Introduction

To circumvent plant immobility, and escape before the plant dies, the majority of plant viruses rely on mobile vectors for their dissemination. Among these vectors, phloem-feeding aphids are by far the most prevalent vectors that can transmit almost half of the insect-borne plant viruses (Hogenhout et al., 2008; Dedryver et al., 2010). Different modes of virus transmission have been described. The non-persistent and non-circulative mode of transmission relies on a transient and brief retention of virions at specific sites in the vector's mouthparts, or in close proximity. In contrast, the persistent, circulative and non-propagative mode of transmission requires endocytosis of virions into aphid cells (Ng and Falk, 2006; Hogenhout et al., 2008; Ammar el et al., 2009; Blanc et al., 2014; Gray et al., 2014; Drucker and Then, 2015; Whitfield et al., 2015). In the latter mode of transmission, viruses may persist in the aphid's body during the whole insect life with (propagative mode) or without (non-propagative mode) replication.

Members of the *Luteoviridae* family (referred to as luteovirids) are single-stranded RNA positive strand viruses, phloem-limited and strictly transmitted by aphids in a circulative, persistent, and non-propagative mode (Gildow, 1999; Gray and Gildow, 2003; Brault et al., 2007). Luteovirid transmission is highly specific because each virus species is usually transmitted efficiently by only one or a few aphid species (Herrbach, 1999). Luteovirid particles are acquired during the prolonged sap ingestion by aphids while feeding on infected plants. Virions are then transported through the gut cells via a transcytosis mechanism that is thought to be initiated by clathrin-mediated endocytosis (Gildow, 1999; Brault et al., 2007). Virus uptake into intestinal cells occurs either at the posterior midgut, the hindgut or both, depending on the virus species (Brault et al., 2007). Once released into the hemocoel, virions may be protected from degradation by binding to symbionin, an endosymbiotic protein, but this interaction, as well as its implication in luteovirid transmission, remains controversial (van den Heuvel et al., 1994, 1997; Filichkin et al., 1997; Liu et al., 2009; Bouvaine et al., 2011; Cilia et al., 2011). From there, luteovirid particles reach the accessory salivary glands (ASG) and are transported through the ASG cells by a transcytosis mechanism before being released into the salivary canal (Brault et al., 2007). These transcytosis events are suspected to rely on the presence of membrane virus-specific receptors at the gut and ASG levels. The apical plasmalemma of the intestinal cells together with the basal lamina and the basal plasmalemma of the ASG cells have been identified as luteovirid transmission barriers in aphids suggesting that specific interactions between virus structural proteins and cellular partners must exist at these locations to allow virus transmission (Gildow, 1999).

Luteovirid capsids are composed of two structural viral proteins namely the major coat protein (CP of about 22 kDa) and the minor capsid protein (readthrough protein or RT^*^ of about 55 kDa) which is not required for virus particle assembly. Both proteins are involved in aphid transmission. Some mutations in the CP sequence of luteovirids affected aphid transmission without impacting virion formation (Torrance, 1992; Brault et al., 2003; Kaplan et al., 2007; Doumayrou et al., 2016). Point mutations or deletions in the RT^*^ are deleterious for efficient transport of virions through the gut cells (Gildow et al., 2000; Reinbold et al., 2001) and for virus uptake into the ASG cells (Brault et al., 1995, 2000; Chay et al., 1996; Bruyère et al., 1997; Peter et al., 2008). Moreover, RT^*^ is responsible for luteovirid vector specificity (Brault et al., 2005).

Identifying luteovirid cellular partners, and in particular virus receptors in aphids, is a major challenge that could ultimately result in the development of innovative technologies aimed at inhibiting virus transmission. Up to now only insecticide treatments and aphid- or virus-tolerant or -resistant plants can be deployed to control luteovirid diseases (Walkey and Pink, 1990; Dogimont et al., 1996; Barker and Waterhouse, 1999; Dreyer et al., 2001). The gut membrane protein alanyl aminopeptidase N (APN), was identified previously as a potential receptor of pea enation mosaic virus (PEMV, *Enamovirus* genus, *Luteoviridae* family) in the aphid species *A. pisum* using an array of *in vitro*-based techniques and insect cells experiments (Linz et al., 2015). APN was isolated from a phage display peptide screen and evidence of its role in PEMV transmission by *A. pisum* was provided by competition experiments between the virus and a peptide potentially mimicking the viral determinant binding to the aphid receptor (Liu et al., 2010). Other aphid proteins exhibiting the ability to bind purified luteovirids *in vitro* have been reported but their precise role in virus transmission has not been identified. This includes several proteins extracted from *Myzus persicae* or heads of *Sitobion avenae* which exhibited the capacity to bind to virions of *Turnip yellows virus* (TuYV), previously designated *Beet western yellows virus* (Seddas et al., 2004), or of *Barley yellow dwarf virus* (BYDV) (Li et al., 2001). Another uncharacterized protein located in the ASG cells of *S. avenae* and *Schizaphis graminum* has also been suspected to be involved in luteovirid transmission, as acquisition of antibodies directed against this protein together with BYDV greatly reduced virus transmissibility (Wang and Zhou, 2003). Finally, by coupling quantitative proteomics with aphid genetics, several proteins from *S. graminum*, including a luciferase and a cyclophilin-like protein, were associated with the ability to transmit *Cereal yellow dwarf virus* (CYDV) (Yang et al., 2008). Subsequently, differential gel electrophoresis (DIGE) coupled to mass spectrometry on an F2 population originating from a cross between vector and non-vector biotypes of *S. graminum* exhibiting different barriers to transmission of CYDV-RPV (gut or ASG) revealed eight proteins under-represented in genotypes with a strong gut barrier (Cilia et al., 2011). Genetics studies of BYDV and CYDV transmission by aphids inferred that transmission capacity is a multigenic trait with some of the aphid genes being shared by the two viruses and some others being specific for one virus species (Papura et al., 2002; Dedryver et al., 2005; Burrows et al., 2006, 2007). Importantly, the aphid proteins predicted to function at specific transmission barriers were expressed as two isoforms with distinct charges (Papura et al., 2002; Cilia et al., 2011). Although the aforementioned studies have identified potential luteovirid partners in aphids, they did not link these proteins to the virus aphid-transmission phenotype.

We performed a yeast two-hybrid screen of a total *M. persicae* cDNA library to identify interactions between aphid cellular components and the structural proteins of the *Cucurbit aphid borne yellows virus* (CABYV) (*Polerovirus* genus, *Luteoviridae* family), which is transmitted efficiently by *M. persicae*. The membrane ephrin receptor (Eph) was identified as a potential binding partner of the RT^*^s of CABYV and TuYV, another polerovirus efficiently transmitted by this aphid species. Functional validation tests were conducted with TuYV in *M. persicae* using techniques based on RNA interference. Feeding aphids on various sources of dsRNA targeting *Eph*-mRNA resulted in reduced internalization of TuYV genomes into the aphid's body and reduced transmission of TuYV, without affecting aphid's fitness. Taken together, these results implicate Eph in the transmission process of TuYV and suggest involvement of this protein in transmission of other poleroviruses by *M. persicae*.

## Materials and methods

### Aphid library construction

Total RNA was isolated from 35 mg of all instars of *M. persicae* using the RNeasy Plant Mini Kit (Qiagen) following the RNeasy Fibrous Tissue Mini Kit protocol. The purification of poly A^+^ RNA from total RNA was performed with the Oligotex™ mRNA Mini Kit (Qiagen) following the Batch protocol. The reverse transcription was performed starting from 1 μg of mRNA using an Oligo(dT)_20_ primer with an adaptor extension and following the MMLV High Performance Reverse Transcriptase procedure (EPICENTRE® BIOtechnologies). The protocol was modified by the addition of a template switching primer (adaptor) after 30 min of incubation for a 3′ cDNA extension (Table S1). The cDNA molecules were amplified using a single adaptor primer with the GoldStar® DNA polymerase (Eurogentec). The cycles were as follows: 95°C 1 min, 95°C 15 s, 65°C 20 s, 72°C 3 min (23 cycles), and 65°C 20 s, 72°C 6 min. The cDNA fragments were further purified with QIAquick PCR Purification Kit (QIAGEN) and subjected to an over-night digestion at 50°C with the restriction enzyme SfiI. Alongside, the pGADT7 vector (Clontech) was modified by the introduction of SfiI sites using a specific pair of complementary oligonucleotides with NcoI and EcoRI sites (Table S1) leading to pGADT7-SfiI. This plasmid was digested with SfiI before being dephosphorylated using the Thermosensitive Alkaline Phosphatase (Promega). A short run electrophoresis of SfiI-cDNA digestion products was performed on low melting point agarose gel to collect the DNA fragments above 400 bp. DNA fragments were recovered after hot-phenol extraction and DNA precipitation. Insert ligation into pGADT7-SfiI was performed at 16°C for 8 h with a molar ratio vector:insert of 1:30. The ligation mixture was further introduced by electroporation into *Escherichia coli* XL10-Gold Ultracompetent Cells (Stratagene, Agilent Technologies). After streaking the bacteria on LB agar containing ampicillin for 19 h at 37°C, colonies were collected in liquid LB medium complemented with glycerol solution (25% final) and stored at −80°C. The cDNA library was then amplified by inoculating 100 ml of LB containing ampicillin with an aliquot of the glycerol stock and cultivating the bacteria for 3 h at 37°C. Plasmids were then purified using QIAfilter Plasmid Maxi Kit (QIAGEN) and used in the yeast two hybrid screen.

### Viral constructs for the yeast two hybrid screen

The major (CP) and the minor (RT^*^) capsid proteins of TuYV (NC_003743) and CABYV (NC_003688) were used in the yeast two hybrid experiments. The CP sequences of TuYV and CABYV were amplified by PCR (Expand High Fidelity PCR System, Roche Applied Science) using appropriate primers (Table S1) from the full-length viral sequences described in Veidt et al. (1992) and Guilley et al. (1994). To clone the RT^*^ sequence of TuYV and CABYV, two overlapping mutagenic oligonucleotides were used in the PCR reaction together with external primers (Table S1) to replace the CP-stop codon by a tyrosine codon in the TuYV and CABYV sequences. The 5′-terminal nucleotide of TuYV- and CABYV-RT^*^ sequence was positioned, respectively, at nt 4793 and nt 4896 on the viral genomes. After digestion with the appropriate restriction enzymes (Promega), CP and RT^*^ from CABYV were purified on column (MSB® Spin PCRapace, Invitek GmbH) and cloned downstream the GAL4 DNA binding domain (BD) into the pGBKT7 vector (Clontech). The CP sequence from CABYV was also introduced downstream the GAL4 activation domain (AD) into the pGADT7 vector (Clontech). In addition, CP, RT^*^ from CABYV together with CP and RT^*^ from TuYV were introduced into pLexA-N vector downstream the LexA binding domain (Dualsystems Biotech). Ligations were performed overnight at 16°C in a 10 μL final volume using a molar ratio vector:insert of 1:5 with the T4 DNA Ligase from Promega. Ligation products were introduced by heat-shock into *E. coli* XL10-Gold competent cells.

The recombinant constructs were referred to as pGBKT7-CP_CA_, pGADT7-CP_CA_, pGBKT7-${\text{RT}}_{\text{CA}}^{*}$, pLexAN-CP_CA_, pLexAN-${\text{RT}}_{\text{CA}}^{*}$, pLexAN-CP_Tu_, and pLexAN-${\text{RT}}_{\text{Tu}}^{*}$. The pGBKT7-derived plasmids were introduced into the Y2HGold yeast strain and the pLexAN-derived plasmids were introduced into the NMY51 yeast strain. Y2HGold and NMY51 yeast strains contain the reporter genes *HIS3* and *ADE2*.

### Yeast two hybrid assays

The *M. persicae* cDNA library was screened against the baits following the procedures described in the DUALhunter kit user manual (Dualsystems Biotech). The colonies were plated onto a stringent medium lacking leucine, tryptophan, histidine, and adenine [-LWHA] and cultivated at 28°C for 3–14 days. The colonies developing on the [-LWHA] medium were selected and the recombinant pGADT7 plasmid containing the aphid cDNA was recovered following a yeast DNA extraction method. Briefly, yeast cells from a 2 ml overnight culture were suspended in 100 μl of a buffer (67 mM Potassium Phosphate, pH 7.5) containing 50 units of Lyticase (L2524, Sigma-Aldrich) and incubated 1 h at 37°C before proceeding with the common alkaline lysis *E. coli* plasmid purification. Recombinant plasmids were introduced into *E. coli* to obtain a sufficient amount of plasmids for sequencing.

### Aphid acquisition of dsRNA from transgenic *A. thaliana* and from *in vitro*-synthesized dsRNA

*Arabidopsis thaliana* expressing a hairpin RNA targeting *Eph* or *LacZ* as a control (Ara:Hp-Eph and Ara:Hp-LacZ) were described in Mulot et al. (2016) and were grown in an environment-controlled chamber at 23°C day and 20°C night with a 10 h photoperiod as well as Col-0 non-transformed plants. *In vitro*-synthesized dsRNA targeting *Eph* or *LacZ* (dsRNA_Eph_ and dsRNA_LacZ_) were obtained as described previously in Mulot et al. (2016).

*M. persicae* (Sulzer) colonies were reared on pepper (*Capsicum annuum*) at 20°C with a 16 h photoperiod. Aphids were fed on transgenic *A. thaliana* or artificially on *in vitro*-synthesized dsRNA as described in Mulot et al. (2016) except that the acquisition time on the artificial medium containing the dsRNA was extended to 5 days in some experiments and the final dsRNA concentration in the feeding medium was set up to 400 ng/μl in all experiments. When a 5-day acquisition period was performed, the dsRNA-containing medium was replaced after 3 days by a fresh medium containing the dsRNA.

### Virus transmission by *M. persicae*

In the virus transmission experiments, aphids previously fed for 10 days on transgenic *A. thaliana* (Ara:Hp-Eph or Ara:Hp-LacZ) were transferred for 24 h on purified TuYV prepared as described in Van den Heuvel et al. (1991). The viral concentration was set up at 25 μg/ml in the artificial diet (Bruyère et al., 1997). Aphids fed artificially on dsRNA were either transferred onto purified virus (same set-up as described above) or on TuYV-infected *M. perfoliata* inoculated by agroinfiltration as described in Hipper et al. (2014). After a 24 h acquisition access period of the virus, two potentially viruliferous aphids were transferred on Col-0 test plants for 72 h. After this inoculation access period, some aphids were collected for further analysis (see below) while the remaining aphids were eliminated by an insecticide treatment. The plants were tested by DAS-ELISA 3 weeks later using virus-specific antibodies as described in Bruyère et al. (1997). In this assay, samples from several young leaves were collected on each plant and pooled before grinding.

### Eph-mRNA and viral RNA accumulation analysis in aphids by real-time reverse transcription polymerase chain reaction (qRT-PCR)

Total RNA was extracted from whole *M. persicae* (20 aphids per sample) as described in Mulot et al. (2016). Total RNA was also extracted from 100 dissected guts using the RNeasy Plant Mini Kit (QIAGEN) as described in Mulot et al. (2016) or alternatively from 35 dissected guts using NucleoSpin® RNA XS (Macherey-Nagel). To evaluate *Eph*-mRNA accumulation, qRT-PCR was performed as in Mulot et al. (2016). As mentioned in Mulot et al. (2016), the relative expression levels were normalized to *Rpl7* and *L27*. To determine the copy number of TuYV genomes internalized into *M. persicae*, total RNA was extracted from whole aphids after transferring them for 3 days on non-infected Col-0 (inoculation access period or IAP) to clear the gut lumen. The viral RNA was converted into cDNA using the reverse primer BPqtR1 and the M-MLV reverse transcriptase kit (Promega). The forward primer BPqtF0 and the reverse primer BPqtR1 (Table S1) were used to amplify by real-time PCR (qRT-PCR) the cDNA corresponding to nts 3694–3830 on TuYV genomic sequence (accession number NC_003743) using the same set-up as described in Mulot et al. (2016). Alongside, viral RNA genomes were extracted from purified virions using the RNeasy Plant Mini Kit (QIAGEN). After quantification at 260 nm (Nanodrop 2000; Thermo Fischer Scientific), the viral RNA was converted to cDNA as described above. Dilution series of 10^9^ to 10^4^ viral cDNA copies obtained from RNA extracted from purified virions were used to calibrate the CFX cycler and comparison between calibrate standard Ct values and samples Ct values provided an absolute quantification of TuYV genomes.

### Aphid fecundity and feeding behavior tests

Aphid fecundity after feeding on transgenic *A. thaliana* (Ara:Hp-Eph or Ara:Hp-LacZ) was assessed by depositing individual fourth instars or adults onto these plants for 2 days. After this period, only one nymph was kept on the plant for 10 days to reach the adult stage before being transferred individually onto non-transformed Col-0 plants. Nymph production was recorded after 5 days. Aphid fecundity was also recorded after feeding fourth instars or adults for 5 days on *in vitro*-synthesized dsRNA. Four aphids were then transferred onto each non-transformed Col-0. Nymph production was monitored during 5 days. A Student *t*-test was applied to the values after controlling that the data followed a linear model.

To evaluate the feeding activity of aphids, fourth instars or adults *M. persicae* fed for 5 days on *in vitro*-synthesized dsRNA, were transferred to an artificial feeding medium (MP148, Harrewijn, 1983) for 48 h. Pools of 9–10 aphids were enclosed in individual boxes (10 or 11 boxes per condition) that were internally covered with a pH-indicator paper prepared in 0.2% bromocresol green dissolved in ethanol. The number of honeydew droplets produced by the aphids and which appear as purple dots on the indicator paper was counted manually or evaluated by image analysis (ImageJ). After determining that the data followed a linear model and variance was equal between samples, a Student *t*-test was applied to the values.

## Results

### The ephrin receptor protein from *M. persicae* is a potential partner of polerovirus structural proteins

In order to identify partners of polerovirus particles in the aphid *M. persicae*, we looked for cellular partners of the structural proteins of CABYV which is efficiently transmitted by this aphid species (Lecoq et al., 1992). An aphid cDNA library was obtained from mRNA extracted from whole aphids. The cDNA library was cloned into the pGADT7 vector and the average insert size was about 400 bp. The major and the minor capsid protein sequences of CABYV (CP_CA_ and ${\text{RT}}_{\text{CA}}^{*}$) were introduced into the pGBKT7 vector and expressed as fusion proteins with the GAL4 DNA binding domain (BD) in the Y2HGold yeast strain in which the *HIS3* and *ADE2* reporter genes are under the control of the GAL4 promoter. Screening the *M. persicae* cDNA library with the baits was performed by introducing the cDNA library into the yeast cells previously transformed with each bait. 6.2 × 10^6^ and 4.6 × 10^6^ double transformed yeast cells were obtained for the CP_CA_ and ${\text{RT}}_{\text{CA}}^{*}$ screens, respectively. When plated onto the [-LWHA] medium to select yeast cells in which *in vivo* interactions occurred, 171 and 5 colonies developed for the CP_CA_ and ${\text{RT}}_{\text{CA}}^{*}$ screens, respectively. A similar cDNA prey sequence, encoding a 244 amino acid peptide, was found in 4 of 13 colonies analyzed for the CP_CA_ screen and in 3 of the 5 colonies which emerged from the ${\text{RT}}_{\text{CA}}^{*}$ screen. When blasted on the *M. persicae* genome (*M. persicae* clone G006 assembly v2, blast server, Aphidbase.com), one scaffold (MYZPE13164 G006 v1.0 000015980) contained the identified sequence which is annotated as ephrin type-B receptor 1-B (LOC111037473) and referred thereafter in the document as Eph (Figure S1).

Ephrin receptors are activated upon binding to their membrane-associated ephrin ligands and plays important roles in developmental processes in mammalian and in pathological diseases like brain and lungs cancers (for review see Pasquale, 2005; Himanen et al., 2007; Genander and Frisen, 2010; Pitulescu and Adams, 2010; Perez White and Getsios, 2014; Kania and Klein, 2016). Interaction of Ephs with ephrin ligands on the surface of neighboring cells triggers Eph kinase-dependent signaling in a bidirectional process. Ephrin receptor family is divided into two subclasses, EphA and EphB, based on amino acid sequence homology and binding affinities to glycosylphosphatidylinositol (GPI)-linked ephrin-A or transmembrane ephrin-B ligands. Interestingly, Eph or ephrin ligand have been shown to display receptor functions for mammalian viruses, bacteria and protozoan parasites (Bonaparte et al., 2005; Lupberger et al., 2011; Kaushansky et al., 2015; Subbarayal et al., 2015).

**Figure 1:**
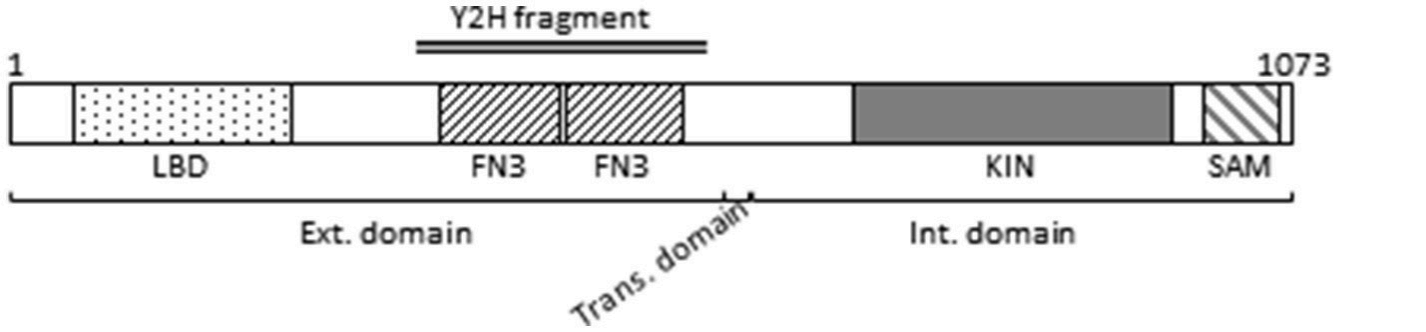
Ephrin receptor schematic representation. The different boxes represent the following domains: LBD: Ephrin receptor Ligand-Binding Domain; FN3: Fibronectin type-III domain; KIN: Protein kinase domain; SAM: Sterile alpha motif domain. The external (Ext.), transmembrane (Trans.), and internal (Int.) domains are indicated. Numbers above the representation stand for amino acids. The double line represents the amino acid sequence encoded by the cDNA clone identified by the yeast two hybrid (Y2H) screen.

The extracellular domain of Ephs contains a globular ligand-binding domain and two fibronectin type III repeats (Figure 1). A short transmembrane domain separates the extracellular part from the intracellular cytoplasmic part consisting of the protein kinase domain and a sterile alpha motif domain responsible for Eph clustering (Stapleton et al., 1999) (Figure 1). The identified candidate peptide from the *M. persicae* cDNA library covers the two fibronectin type III repeats (Figure 1).

**Figure 2:**
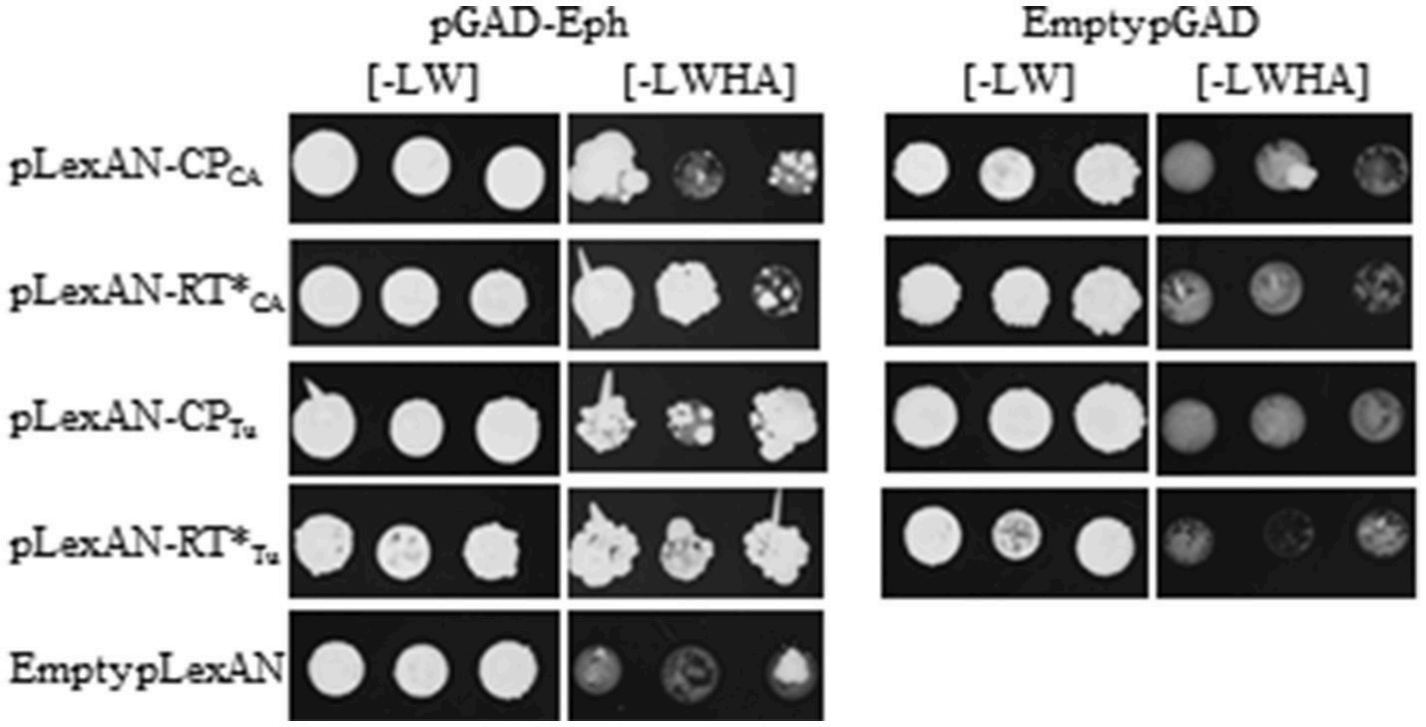
Interaction between CABYV and TuYV structural proteins and Eph partial domain. The yeast strain NMY51 was co-transformed with pGAD-Eph and one of the following constructs: pLexAN-CP_CA_, pLexAN-RT^*^_CA_, pLexAN-CP_Tu_, pLexAN-RT^*^_Tu_, or the empty pLexAN. In parallel, yeast cells were co-transformed with the empty pGAD and one of the viral pLexAN-derived plasmids mentioned above. Three colonies were allowed to grow on a medium lacking leucine and tryptophan [-LW] before being transferred onto a selective medium deprived of leucine, tryptophan, histidine and adenine [-LWHA]. Yeast cells were grown at 28°C for 3 days on [-LW] and for 14 days on [-LWHA] media.

To address the ability of the plasmid bearing the partial *Eph* cDNA sequence (referred to hereafter as pGAD-Eph) to activate by itself the transcription of the reporter genes (*HIS3* and *ADE2*), the pGAD-Eph plasmid was introduced together with the empty pGBKT7 vector into Y2HGold yeast strain in which the reporter genes were controlled by the GAL4-responsive promoter. The yeast double-transformed colonies were plated onto medium lacking histidine [-LWH] or histidine and adenine [-LWHA]. Yeast growth was observed on both medium showing the capacity of the Eph partial domain to activate transcription of the reporter genes in the absence of luteovirid CP or RT^*^ (Figure S2). Interaction of the Eph domain with the GAL4-promoter can be considered as a false positive reaction, but could also mask a true interaction with the viral baits. Considering the function of Eph as human virus receptors, we pursued the yeast two hybrid binding assays and addressed whether the Eph domain was able to interact with another promoter, the LexA promoter. Interestingly, no autoactivation of the transcription of the reporter genes *HIS3* and *ADE2* was observed when the pGAD-Eph and the empty pLexAN plasmids were introduced into the NMY51 yeast strain in which the reporter genes are under the control of the LexA promoter and when the doubled-transformed cells were plated onto [-LWHA] medium for 7 days (Figure S3). A low yeast growth was however observed, in one out of the three colonies, when the growth was extended to 14 days (Figure 2), implying that a low yeast development should not be considered as a true interaction between the prey and baits.

Therefore, the interactions between CABYV baits and the Eph partial domain were confirmed using the NMY51 yeast strain. The viral structural protein sequences were cloned into the pLexAN yeast vector as fusion proteins with the LexA DNA binding domain (BD). The resulting plasmids referred to as pLexAN-CP_CA_ and pLexAN-${\text{RT}}_{\text{CA}}^{*}$ were introduced into the NMY51 yeast strain together with the pGAD-Eph. Three colonies of the double-transformed yeast cells were plated onto the [-LW] to control yeast growth and on the [-LWHA] stringent medium to select yeast cells in which *in vivo* interaction occurred. We observed that ${\text{RT}}_{\text{CA}}^{*}$ was able to interact with the partial domain of Eph since 2 out of 3 colonies developed on the [-LWHA] medium after 14 days of growth. Interaction between the Eph domain and CP_CA_ was less clear as only one out of the 3 colonies grew well on the [-LWHA] medium after 14 days (Figure 2). To control whether Eph could be a potential partner of other polerovirus structural proteins, we tested TuYV, which is also transmitted efficiently by *M. persicae* (Leiser et al., 1992). The TuYV CP and RT^*^ sequences were introduced into the pLexAN vector leading to the pLexAN-CP_Tu_ and pLexAN-${\text{RT}}_{\text{Tu}}^{*}$. Similarly as described above, the pLexAN recombinant plasmids were introduced into NMY51 yeast cells together with pGAD-Eph and plated onto the [-LWHA] medium. Interestingly, interaction of the Eph domain with the ${\text{RT}}_{\text{Tu}}^{*}$ was clearly observed (all the three colonies developed well on the [-LWHA] medium after 14 days of growth) and an interaction with the CP_Tu_ was also suggested since two out of the three colonies grew on the selective medium (Figure 2). The reason for the uneven growth of the three double-transformed colonies on the stringent medium is unknown but is likely due to a tendency of the CP-fusion proteins to self-assemble in yeast (Figure S4) rather than to interact with the prey. No yeast growth was observed on the [-LWHA] medium when the viral pLexAN-derived plasmids were co-transformed with the empty pGAD vector (Figure 2). Binding of Eph domain to the TuYV baits were controlled in an additional experiment (Figure S3). No yeast growth was observed for the control combinations.

In conclusion, we observed an unambiguous interaction between the Eph domain isolated from the *M. persicae* cDNA library and the RT^*^ from TuYV (${\text{RT}}_{\text{Tu}}^{*}$). In contrast, interaction of Eph domain with the RT^*^ (${\text{RT}}_{\text{CA}}^{*}$) and the CP from CABYV (CP_CA_) and from TuYV (CP_Tu_) was less clear due to uneven or low growth of the double transformed yeast cells on the selective medium.

### Feeding *M. persicae* on transgenic *A. thaliana* expressing dsRNA_Eph_ or on *in vitro*-synthesized dsRNA_Eph_ reduces aphid's ability to transmit TuYV

We first analyzed the function of Eph in TuYV transmission by *M. persicae* since a clear interaction was observed in yeast between the Eph domain picked up from the yeast two hybrid screen and ${\text{RT}}_{\text{Tu}}^{*}$. A way to address whether Eph could be involved in TuYV transmission by *M. persicae* is to silence Eph expression in aphids and evaluate the ability of the modified aphids to transmit the virus. We previously compared five different techniques based on the ingestion by aphids of dsRNA molecules targeting *Eph* (Mulot et al., 2016). We selected the two most efficient techniques i.e., feeding aphids (i) on transgenic plants expressing an RNA hairpin of 249 bp corresponding to a central sequence of *Eph* (Hp-Eph) or (ii) on *in vitro*-synthesized dsRNA of similar sequence. This sequence did not share any sequence identity more than 16 bp with other *M. persicae* expressed genes.

*M. persicae* were first fed for 10 days on T1 lines of transgenic *A. thaliana* expressing the Hp-Eph. Acquisition of dsRNA_Eph_ and/or siRNA_Eph_ from transgenic plants reproducibly inhibited accumulation of *Eph*-mRNA in whole aphids (53–61% reduction of *Eph*-mRNA accumulation in two independent experiments) when compared with aphids fed on control plants (transgenic *A. thaliana* expressing a 276 bp hairpin RNA targeting the bacterial gene *LacZ*, Hp-LacZ) (Table 1). Depending on the dsRNA sources (Hp-Eph or Hp-LacZ), the aphids were thereafter referred to as dsRNA_Eph_-treated aphids or dsRNA_LacZ_-treated aphids. After the dsRNA acquisition from plants, aphids were transferred onto an artificial medium containing purified virus for 24 h. After feeding on virus, the potentially viruliferous dsRNA-treated aphids were then deposited onto Col-0 test plants for virus inoculation and infection of the test plants was assessed by DAS-ELISA 3 weeks later. Interestingly, viruliferous dsRNA_Eph_-treated aphids transmitted TuYV with a significantly lower efficiency than dsRNA_LacZ_-treated aphids (Table 1). Moreover, the lower ability of the viruliferous dsRNA_Eph_-treated aphids to transmit TuYV was correlated with a statistically reduced accumulation of the viral genomes (6–11 times less) in the dsRNA_Eph_-treated aphids when compared to dsRNA_LacZ_-treated aphids (Table 1).

**Table 1:** Effect of dsRNA_Eph_ acquisition from transgenic *A. thaliana* on TuYV transmission by *M. persicae* and genome internalization in aphids.

**Exp**.	**Aphid feeding source^a^**	**AAP^b^**	**Relative fold change *Eph*-mRNA in whole *M. persicae^c^***	**Source for virus acquisition^d^**	**nb inf/inoc plants^e^**	**% of transmission inhibition^f^**	**Virus genome copies/μg RNA internalized into aphids^g^**
1	Ara:Hp-Eph (T1) line 1	10 d	0.40 ± 0.11^*^ (−60%) *1.7E-02*0.45 ± 0.04^*^ (−55%)*2.6E-02*0.39 ± 0.16^*^ (−61%)*1.4E-02*	TuYV 50 ng/μl	6/31^*^ (19%) *9.0E-06*	77	3.72 × 10^6^ ± 7.33 × 10^4^^*^ 2.8E-06
	Ara:Hp-LacZ (T1) line 1		1.00 ± 0.19		16/19 (84%)		2.22x10^7^ ± 8.35 × 10^5^
2	Ara:Hp-Eph (T1) line 1	10 d	0.47 ± 0.04^*^ (−53%)*2.5E-02*	TuYV 50 ng/μl	7/19^*^ (37%)*1.4E-04*	61	1.76 × 10^7^ ± 1.14 × 10^6^*^^ 1.5E-06
	Ara:Hp-Eph (T1) line 2		0.42 ± 0.03^*^ (−58%) *8.6E-04*		7/13^*^ (54%)*8.4E-03*	43	1.39 × 10^7^ ± 6.69 × 10^5^*^^ 1.3E-06
	Ara:Hp-LacZ (T1) line 1		1.00 ± 0.02		19/20 (95%)		1.55 × 10^8^ ± 5.21 × 10^6^

a *Transgenic A. thaliana expressing an hairpin RNA targeting Eph (Hp-Eph) or LacZ (Hp-LacZ)*.

b *Acquisition Access Period for dsRNA acquisition; d = days*.

c Relative fold change of Eph-mRNA accumulation ± standard deviation of triplicates. In brackets the level of expression compared to aphids fed on control conditions (A. thaliana expressing dsRNA targeting LacZ). Each result corresponds to one pool of 20 aphids. ^*^ and bold characters indicate significant differences in accumulation of Eph-mRNA (Student t-test, p < 0.05; p-values are indicated in italics).

d *Virus acquisition was 24 h and virus inoculation 7 days on Col-0 test plants. Five viruliferous aphids were deposited per test plant*.

e Number of plants positive by DAS-ELISA/total number of plants inoculated with aphids. ^*^ and bold characters indicate significant differences in the number of plants infected between both conditions (Fisher's exact test, p < 0.05; p-values are indicated in italics). In brackets, the percentage of infected plants.

f *Percentage of TuYV transmission inhibition when using dsRNA_Eph_-treated aphids when compared to dsRNA_LacZ_-treated aphids. The percentage of infected plants for the LacZ control condition was considered as 100%*.

g TuYV genome copies analyzed by qRT-PCR in whole aphids (pool of 20 aphids) collected after the 4 days of the inoculation access period on the test plants. ^*^ and bold characters indicate significant difference in the amount of viral genomes between the two conditions (Student t-test, p < 0.05; p-values are indicated in italics).

To confirm these results, *M. persicae* were fed artificially on *in vitro*-synthesized dsRNA targeting *Eph*, since this technique reduces *Eph*-mRNA accumulation in the aphid gut (Mulot et al., 2016). In the first two experiments, using an experimental set-up (72 h AAP on dsRNA-Eph at 200 or 400 ng/μl) described in Mulot et al. (2016), we observed a reduction in the accumulation of *Eph*-mRNA in the aphid gut (53 and 20% inhibition of *Eph*-mRNA accumulation in guts) (Table 2, Exp. 1 & 2). However, no reduction in TuYV transmission efficiency was observed after feeding the dsRNA_Eph_-treated aphids on purified virus (Table 2, Exp. 1 and 2). While maintaining the dsRNA concentration in the artificial feeding medium at 400 ng/μl, the acquisition time was then extended to 5 days and the virus transmission assay was performed as before. In three independent experiments (Table 2, Exp. 3–5), a significant reduction in TuYV transmission efficiency (from 38 to 81% of reduction) by the dsRNA_Eph_-treated aphids was observed. Again, the reduction in the virus transmission efficiency was correlated with a significant lower TuYV genome accumulation in the dsRNA_Eph_-treated aphids after gut clearing (Table 2, Exp. 4 & 5). In these two experiments, TuYV accumulated about 4 times less in the dsRNA_Eph_-treated aphids compared to dsRNA_LacZ_-treated aphids. In order to see whether the nature of the virus source could impact the virus transmission efficiency of the dsRNA_Eph_-treated aphids, virus acquisition was performed on TuYV-infected *Montia perfoliata*. Again, TuYV transmission rate was reduced by 50 and 47% when using the dsRNA_Eph_-treated aphids (Table 2, Exp. 5 & 6) although the difference in virus transmission was not statistically significant compared with dsRNA_LacZ_-treated aphids. Nevertheless, this reduction in the TuYV transmission efficiency by the dsRNA_Eph_-treated aphids correlated with a statistically significant decrease of viral genomes internalized (1.3- and 2.3-fold fewer viral genomes in dsRNA_Eph_-treated aphids than in dsRNA_LacZ_-treated aphids) (Table 2, Exp. 5 & 6).

**Table 2:** Effect of *in vitro*-synthesized dsRNA_Eph_ acquisition on TuYV transmission by *M. persicae* and genome internalization in aphids.

**Exp**.	**Aphid feeding source^a^**	**AAP^b^**	**Relative fold change *Eph*-mRNA in *M. persicae* guts^c^**	**Source for virus acquisition^e^**	**nb inf/inoc plants^f^**	**% of transmission inhibition^g^**	**Virus genome copies/μg RNA internalized into aphids^h^**
1	dsRNA_Eph_	72 h	0.47 ± 0.02^*^ (−53%)^d^ *1.0E-05*	TuYV 25 ng/μl	18/20 (90%)*6.6E-01*	no	*nd*
	dsRNA_LacZ_		1.00 ± 0.03		16/20 (80%)		
2	dsRNA_Eph_	72 h	0.80 ± 0.03^*^ (−20%)*1.5E-02*	TuYV 25 ng/μl	44/69 (64%)*3.2E-01*	no	*nd*
	dsRNA_LacZ_		1.00 ± 0.05		43/79 (54%)		
3	dsRNA_Eph_	5 d	2.78 ± 0.10^*^ (+178%)*4.2E-03*	TuYV 25 ng/μl	15/30^*^ (50%)*1.8E-02*	38%	*nd*
	dsRNA_LacZ_		1.00 ± 0.06		24/30 (80%)		
4	dsRNA_Eph_	5 d	0.20 ± 0.00^*^ (−80%)*2.8E-06*	TuYV 25 ng/μl	10/39^*^ (26%)*1.9E-05*	67%	3.65 × 10^6^ ± 1.25 × 10^5^*^^ 3.1E-06
	dsRNA_LacZ_		1.00 ± 0.05		27/35 (77%)		1.48 × 10^7^ ± 5.03 × 10^5^
5^i^	dsRNA_Eph_	5 d	1.44 ± 0.04^*^ (+44%) *2.3E-04*	TuYV 25 ng/μl	3/30^*^ (10%) *5.0E-04*	81%	1.90 × 10^6^ ± 1.52 × 10^4^*^^ 9.4E-06
	dsRNA_LacZ_		1.00 ± 0.02		15/28 (54%)		8.90 × 10^6^ ± 4.30 × 10^5^
	dsRNA_Eph_		1.44 ± 0.04^*^ (+44%) *2.3E-04*	Inf. TuYV *M. perfoliata*	5/30 (17%)*2.3E-01*	50%	1.76 × 10^6^ ± 4.68 × 10^4^*^^ 3.1E-05
	dsRNA_LacZ_		1.00 ± 0.02		10/30 (33%)		2.36 × 10^6^ ± 1.69 × 10^5^
6	dsRNA_Eph_	5 d	0.91 ± 0.02 (−9%) *1.6E-01*	Inf. TuYV *M. perfoliata*	9/33 (27%)*7.7E-01*	47%	1.81 × 10^6^ ± 4.38 × 10^4^*^^ 4.0E-05
	dsRNA_LacZ_		1.00 ± 0.05		17/33 (52%)		4.22 × 10^6^ ± 2.08 × 10^5^

a *The aphid feeding source were in vitro dsRNA targeting Eph (dsRNA_Eph_) or LacZ (dsRNA_LacZ_) at a concentration of 400 ng/μl in the artificial medium except in Exp. 1 where the concentration was 200 ng/μl*.

b *Acquisition Access Period for dsRNA acquisition; h = hours; d = days*.

c Relative fold change of Eph-mRNA accumulation ± standard deviation of triplicates. In brackets the level of expression compared to aphids fed on control conditions (dsRNA_LacZ_). Each result corresponds to one pool of 100 aphid guts. ^*^ and bold characters indicate significant differences between aphids fed on dsRNA_LacZ_ and aphids fed on dsRNA_Eph_ (Student t-test, p < 0.05; p-values are indicated in italics).

d *Eph-mRNA accumulation from Mulot et al. (2016) (Table 2E, Exp. 1)*.

e *After feeding on dsRNA, aphids were first transferred onto purified virus or infected M. perfoliata for 24 h before being transferred onto Col-0 test plants for 72 h*.

f Number of plants positive by DAS-ELISA/total number of plants inoculated with aphids. ^*^ and bold characters indicate significant differences in the number of plants infected between both conditions (Fisher's exact test, p < 0.05; p-values are indicated in italics). In brackets, the percentage of infected plants.

g *Percentage of TuYV transmission inhibition when using dsRNA_Eph_-treated aphids when compared to dsRNA_LacZ_-treated aphids. no = no reduction of the virus transmission efficiency. The percentage of infected plants for the LacZ control condition was considered as 100%*.

h *TuYV genome copies analyzed by qRT-PCR in whole aphids (pool of 20 aphids) collected after the 4 days of the inoculation access period on the test plants*.

i *In Exp. 5, after dsRNA acquisition, the aphids were split into two batches; one acquired TuYV from purified virus and the other from TuYV-infected plants*.

**Figure 3:**
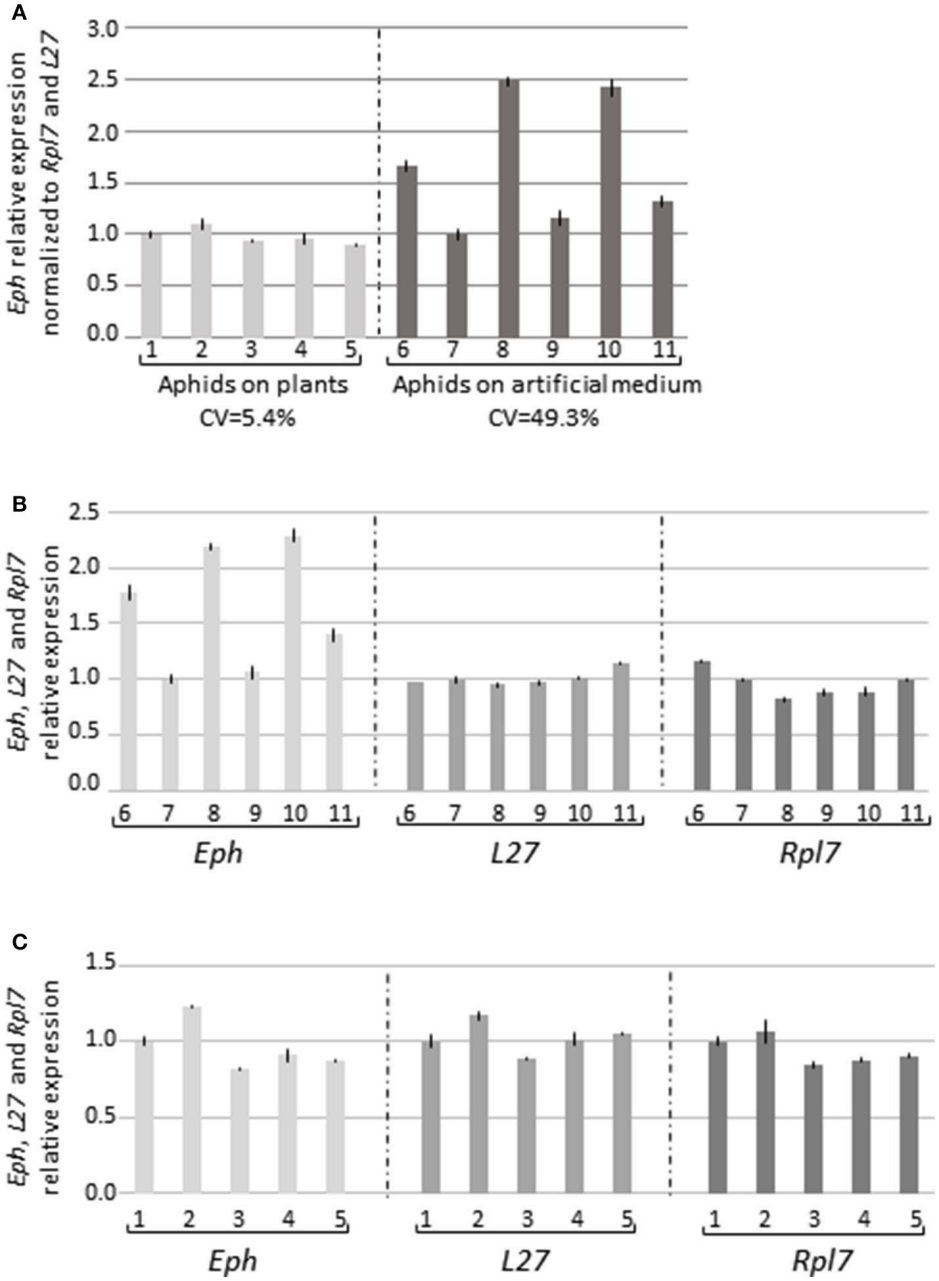
*Eph*-mRNA accumulation in guts from aphids fed on plants or on artificial medium. **(A)** Guts (35 per sample) were dissected from *M. persicae* reared on pepper plants (five samples) or fed during 5 days on the artificial medium MP148 (Harrewijn, 1983) (six samples). The data represent the relative expression of *Eph* in each sample normalized to the accumulation of the two reference genes *L27* and *Rlp7* ± standard deviation of triplicates. The first sample for each condition was arbitrarily fixed to 1. CV: coefficient of variation. *Eph, L27*, and *Rpl7* expression without normalization in aphids fed on an artificial medium **(B)** or on plants **(C)**. Similar numbers referred to the same biological samples.

Surprisingly, the virus transmission reduction observed in the five experiments (Table 2, Exp. 3–6) was not always correlated with a reduction of *Eph*-mRNA accumulation in the gut cells and, in two experiments, a higher accumulation of *Eph*-mRNA was observed in the dsRNA_Eph_-treated aphids compared to dsRNA_LacZ_-treated aphids (Table 2, Exp. 3 & 5). To evaluate whether the artificial feeding step of aphids on an artificial medium for 5 days could affect *Eph* expression stability, we compared *Eph*-mRNA accumulation in the digestive tubes of aphids fed for 5 days on artificial medium with those of aphids fed on *C. annuum* (plant species used to rear *M. persicae*). Unexpectedly, accumulation of *Eph*-mRNA (normalized to the expression of the two housekeeping genes *L27* and *Rpl7*) in gut cells varied significantly when the aphids were fed on artificial medium (Figures 3A, S5). Expression of *L27* and *Rpl7* was however stable in similar conditions (Figure 3B). In contrast, expression of *Eph* as well as *L27* and *Rpl7* was stable in gut samples collected from aphids fed on plants (Figures 3A,C, S5). The high variation of *Eph*-mRNA accumulation in aphids fed on artificial medium may account for our inability to reproducibly observe a reduction of *Eph*-mRNA accumulation in aphids fed during 5 days on an artificial diet containing the dsRNA_Eph_.

### Feeding *M. persicae* on *in vitro*-synthesized dsRNA_Eph_ impacts internalization of other poleroviruses into the aphid's body

The specific reduction of virus transmission after acquisition of dsRNA_Eph_ was evaluated with two other poleroviruses transmitted by *M. persicae* (Lecoq et al., 1992; Stevens et al., 2005), *Beet mild yellowing virus* (BMYV) and CABYV. After *in vitro* acquisition of dsRNA, dsRNA_Eph_-treated aphids transmitted less efficiently BMYV in two independent experiments (38 and 31% transmission inhibition in Exp. 1 and 2, respectively, Table 3), but the transmission inhibition was not statistically significant when compared to the control condition using dsRNA_LacZ_-treated aphids. However, a statistically significant decrease of BMYV genomes internalized into the dsRNA_Eph_-treated aphids was measured (1.8 and 1.4 times less viral genomes in dsRNA_Eph_-treated aphids than in dsRNA_LacZ_-treated aphids; Exp. 1 and 2, respectively, Table 3). When CABYV was used as a virus source, the number of infected plants was particularly low, which makes it difficult to draw a clear conclusion on the effect of dsRNA_Eph_ acquisition on the CABYV transmission efficiency (Table 3, Exp. 3). Such low transmission efficiency of CABYV from purified virus has previously been observed in our laboratory even with high concentrations of virus in the artificial medium (V. Brault, unpublished), and could be explained by instability of the particles after the purification procedure. Nevertheless, a moderate, but still significant, reduction of the number of CABYV genomes internalized was observed in the dsRNA_Eph_-treated aphids (Table 3, Exp. 3). These results suggest that Eph could have a broader role in poleroviruses transmission by *M. persicae*.

**Table 3:** Effect of *in vitro*-synthesized dsRNA_Eph_ acquisition on BMYV and CABYV transmission by *M. persicae* and genome internalization in aphids.

**Exp**.	**Aphid feeding source^a^**	**Source for virus acquisition^b^**	**nb inf/inoc plants^c^**	**% of transmission inhibition^d^**	**Virus genome copies/μg RNA internalized into aphids^e^**
1	dsRNA_Eph_	BMYV 50 ng/μl	10/32 (31%) *1.0E-01*	38%	1.28 × 10^6^ ± 1.25 × 10^5*^ 2.9E-03
	dsRNA_LacZ_		16/32 (50%)		2.30 × 10^6^ ± 2.41 × 10^5^
2	dsRNA_Eph_	BMYV 50 ng/μl	11/28 (39%) *1.4E-01*	31%	3.02 × 10^6^ ± 0.52 × 10^5*^ 7.8E-05
	dsRNA_LacZ_		16/28 (57%)		4.15 × 10^6^ ± 1.06 × 10^5^
3	dsRNA_Eph_	CABYV 100 ng/μl	1/11 (9%) ^f^*2.9E-01*	67%	6.02 × 10^6^ ± 4.46 × 10^5*^ 2.7E-03
	dsRNA_LacZ_		3/11 (27%)		8.11 × 10^6^ ± 3.14 × 10^5^

a *The aphid feeding source were in vitro dsRNA targeting Eph (dsRNA_Eph_) or LacZ (dsRNA_LacZ_) at a concentration of 400 ng/μl in the artificial medium and the AAP was fixed to 5 days*.

b *After feeding on dsRNA, aphids were first transferred onto purified virus for 24 h before being transferred onto Col-0 test plants for 72 h*.

c *Number of plants positive by DAS-ELISA/total number of plants inoculated with aphids. In brackets, the percentage of infected plants. p-values from the Fisher's exact test are indicated in italics*.

d *Percentage of TuYV transmission inhibition when using dsRNA_Eph_-treated aphids when compared to dsRNA_LacZ_-treated aphids. The percentage of infected plants for the LacZ control condition was considered as 100%*.

e BMYV or CABYV genome copies analyzed by qRT-PCR in whole aphids (6 pools of 20 aphids in Exp. 1, 3 pools of 20 aphids in Exp. 2 & 3) collected after the 4 days of inoculation access period on test plants. ^*^ and bold characters indicate significant difference in the amount of viral genomes between the two conditions (Student t-test, p < 0.05; p-values are indicated in italics).

f *In this experiment, four viruliferous aphids (instead of two in the other experiments) were transferred on each test plant for virus inoculation*.

### The inhibition of TuYV transmission by dsRNA_Eph_-treated aphids is not due to a reduction in feeding activity

**Figure 4:**
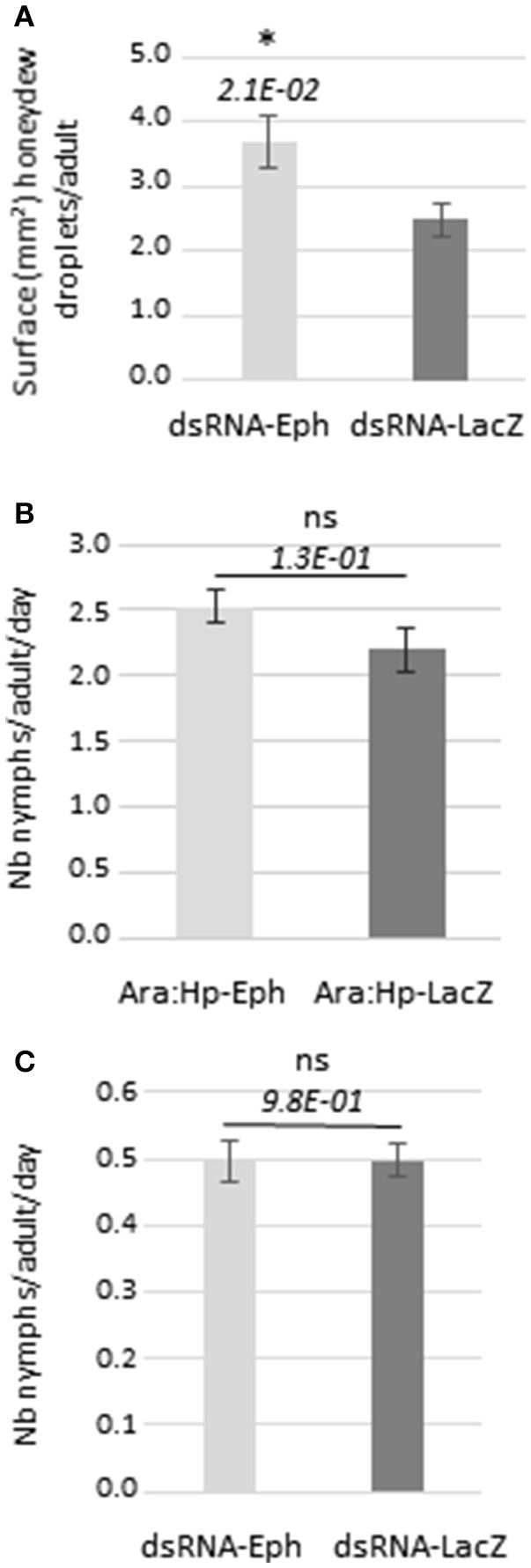
Feeding activity and fecundity of dsRNA-treated aphids. **(A)** Honeydew excretion from dsRNA-treated aphids (after 5 days of acquisition) placed onto feeding artificial medium for 48 h; the bars represent the average of the surface of droplets (surface in mm^2^) produced per adult or nymph during 48 h. *n* = 99 for each condition **(B)** *M. persicae* fecundity after feeding first instars on Ara:Hp-Eph or Ara:Hp-LacZ for 10 days. After this period, individual adults were transferred to non-transformed Col-0 (*n* = 18 for Ara:Hp-Eph and *n* = 10 for Ara:Hp-LacZ) and nymph production was recorded after 5 days. **(C)** *M. persicae* fecundity after feeding fourth instars or adults on *in vitro*-synthesized dsRNA_Eph_ or dsRNA_LacZ_ for 5 days before transferring 4 adults on individual Col-0 (*n* = 28). Nymph production was recorded after 5 days. *n* = 1 12 for each condition. Data from **(B)** and **(C)** are from one experiment and represent the mean value of nymphs produced daily per adult ± standard deviation; ns: non-significant after Student *t*-test (*p*-value > 0.05); ^*^: *p*-value < 0.05 after Student *t*-test. *p*-values are indicated.

The lower virus transmission efficiency of dsRNA_Eph_-treated aphids could be due to a reduced feeding activity on the virus source. Since the majority of the virus transmission experiments presented in this manuscript (9 out of 11 in total) were performed using purified virus as the virus source, we measured the feeding activity of the dsRNA-treated aphids when placed onto the artificial diet. Electropenetrography could not be developed to measure the feeding phases of aphids because this technique is not adapted to evaluate sustained ingestion activity of aphids from an artificial medium (Tjallingii, 1985). Therefore, we measured honeydew excretion of the dsRNA_Eph_-treated aphids after transferring them onto a fresh artificial medium for 48 h. The aphid feeding activity on plants infected with luteovirids has been previously correlated with the efficiency of virus transmission (Sylvester, 1967; Van den Heuvel and Peters, 1990). The surface area covered by honeydew droplets produced by dsRNA_Eph_-treated aphids was slightly higher, than the one secreted by dsRNA_LacZ_-treated aphids (Figures 4A, S6). In another experiment, no difference in honeydew excretion was observed between dsRNA_Eph_- and dsRNA_LacZ_-treated aphids (Figure S7). These results show that the reduction of TuYV transmission by dsRNA_Eph_-treated aphids is not correlated with a lower feeding activity of these aphids on the artificial medium and therefore not linked to a reduced ingestion of virus particles.

### Fecundity of dsRNA_Eph_-treated aphids is not affected by ingestion of dsRNA and/or siRNA from transgenic *A. thaliana*:Hp-Eph or from *in vitro*-synthesized dsRNA_Eph_

In order to investigate the impact of dsRNA and/or siRNA acquisition from transgenic plants or from *in vitro*-synthesized dsRNA on aphid's fecundity, nymph production by the dsRNA-treated aphids was analyzed. Feeding aphids during 10 days on the transgenic plants (Ara:Hp-Eph or Ara:Hp-LacZ) (Figure 4B) or during 5 days on the dsRNA targeting *Eph*-mRNA or *LacZ*-mRNA (Figure 4C) did not affect aphid's fecundity. These results suggest that acquisition of dsRNA or siRNA from either source did not significantly impact aphid physiology. A low fecundity of aphids fed during 5 days on the artificial medium containing the dsRNA can be observed (Figure 4C). The artificial medium composition may affect aphid fecundity but the amount of dsRNA ingested by aphids from the artificial diet may also have an impact on the aphid physiology.

## Discussion

To be transmitted by aphids luteovirids acquired in the phloem of infected plants must cross several epithelia at the gut and salivary gland levels in the vector before being inoculated into a plant during feeding. Virion transport through the epithelia requires virus particle recognition by specific receptors (Mercer et al., 2010). In the present study, we identified Eph as a potential receptor of TuYV in *M. persicae*. Part of the external domain of Eph, corresponding to the fibronectin type III repeats, was able to bind in yeast to the minor capsid protein (RT^*^) of TuYV, which is strictly required for aphid transmission (Brault et al., 1995). RT^*^ protein is involved in the transcytosis of TuYV through the posterior midgut cells and strictly required for virus internalization into the ASG cells (Brault et al., 1995; Reinbold et al., 2001). We showed that feeding *M. persicae* with siRNA/dsRNA targeting *Eph*-mRNA, produced *in planta* or *in vitro*, prior to TuYV acquisition, consistently reduced TuYV accumulation in aphids and subsequently virus transmission to plants. Since siRNA/dsRNA ingestion by aphids did not affect aphid feeding behavior, we concluded that ingestion of siRNA/dsRNA targeting *Eph*-mRNA likely affected TuYV acquisition by *M. persicae*. A lower accumulation of two other poleroviruses, BMYV and CABYV, in similarly treated aphids, was also observed, suggesting a broader implication of Eph in the internalization of poleroviruses into *M. persicae*.

Virus transmission reduction was sometimes correlated with a reduction in the accumulation of *Eph*-mRNA in aphids, in particular after feeding aphids on transgenic plants expressing the dsRNA_Eph_ during 10 days. However, we observed *Eph*-mRNA instability in aphids fed on an artificial medium. This instability could be potentially intensified, in these non-natural conditions, by fluctuations in dsRNA ingestion along the acquisition period, or alternatively, by dsRNA stability that could be altered during this 5-day period. Nevertheless, it is important to mention that virus transmission inhibition was always linked to a reduction of virus accumulation in aphids. Although we show that Eph is involved in polerovirus transmission by *M. persicae*, we cannot conclude from our experiments whether Eph is acting at the gut, at the ASG, or at both levels in *M. persicae*. Nevertheless, considering that oral acquisition of dsRNA targeting Eph preferentially affects gene expression in the gut level (Mulot et al., 2016), and reduces virus accumulation into the aphid's body, it is likely that Eph, at least, is acting at the gut level.

Ephrin receptors are good candidates to be involved in polerovirus transmission by *M. persicae*. In most of the cases, Ephs and their ephrin ligands control a wide array of cell-to-cell interactions in mammals without involving internalization of both proteins. In contrast, in some instances the interaction between Ephs and ligands results in the endocytosis of the complex (Marston et al., 2003; Zimmer et al., 2003; Pitulescu and Adams, 2010), a phenomenon that clearly resembles polerovirus internalization into aphid cells. Moreover, there is evidence that Eph receptors can also be activated by soluble ephrin ligands present in the environment (Alford et al., 2010). In this regard, it is interesting to point out that Eph receptors are involved in human and simian virus uptake. Eph A2 was identified as a host co-factor for Hepatitis C virus entry in liver cells (Lupberger et al., 2011). Internalization of this enveloped virus by clathrin-dependent endocytosis requires several cell-surface molecules, some thought to be essential receptors while others facilitating virus uptake (von Hahn and Rice, 2008). Another human virus, the *Kaposi's sarcoma-associated herpesvirus* (KSHV) also relies on the presence of Eph A2 to enter epithelial cells, but by micropinocytosis rather than endocytosis (Chakraborty et al., 2012; Hahn et al., 2012). Finally, the *Rhesus monkey rhadinovirus*, a closely relative to KSHV, use a wide array of Ephs to be endocytosed into monkey endothelial cells (Hahn and Desrosiers, 2013).

It is also interesting to mention that several Eph receptors are known to bind to caveolin-1, a protein involved in endocytosis of non-enveloped viruses like simian virus 40 (Pelkmans et al., 2001), echovirus 1 (Marjomaki et al., 2002), and *Junonia coenia* densovirus (Wang et al., 2013). Although luteovirids are thought to be internalized into aphid cells by clathrin-mediated endocytosis, an alternative route for virus uptake based on caveolae is still conceivable (Gildow, 1999; Brault et al., 2007). Indeed, compared to clathrin-mediated endocytosis, caveolae entry results in the formation of vesicles that do not enter the traditional acidic endosome/lysosome system (Thomsen et al., 2002). By avoiding acidification, virus internalization into caveolae might be beneficial for non-replicating viruses, like poleroviruses. In the light of these results, it could be interesting to reassess the mechanism of polerovirus internalization into aphid cells by targeting Cav-1 expression in aphids by RNA interference or by using caveolin-specific inhibitors (Rejman et al., 2005).

The ephrin type-B receptor 1-B could be the second aphid protein identified as a potential luteovirid receptor. APN was previously shown to be involved in PEMV (*Enamovirus* genus) internalization into *A. pisum* (Liu et al., 2010; Linz et al., 2015). Here, we bring evidence that Eph is another aphid protein involved in polerovirus acquisition and transmission by *M. persicae*. Determining whether these two proteins act in concert in both aphid species, or are specific for one aphid species, will be a challenge for future studies. As already mentioned, implication of several proteins in the internalization process of mammalian viruses into cells is more likely a general mechanism: viral surface components must first bind to attachment factors on the cell surface before interacting with receptors that drive reactions leading to entry (Mercer et al., 2010; Grove and Marsh, 2011; Cossart and Helenius, 2014). One extreme example is HCV which has been shown to require about ten different molecules for cell entry (Grove and Marsh, 2011). Interestingly, some of them are responsible for virus non-specific attachment on the cell surface while interaction with the liver specific intercellular adhesion molecule-3-grabbing non-integrin (L-SIGN) is thought to confer tissue tropism *in vivo* (Gardner et al., 2003). Analyzing *Eph*-mRNA distribution along the digestive tube of *M. persicae* will show whether this protein is responsible for TuYV gut tropism at the posterior midgut.

In this study, we showed that acquisition by aphids of dsRNA molecules targeting *Eph* can reduce TuYV transmission. Aphid survival and fecundity were not affected by *Eph*-mRNA targeting. Eph may therefore be an ecologically safe target to reduce luteovirids impacts by inhibiting their dispersion by aphids. The dsRNA_Eph_ molecules could be expressed in transgenic plants (as in this study), but could also alternately be sprayed on cultures. This innovative delivery system has been assayed on different aphid species by aerosolizing siRNA targeting a carotene dehydrogenase and a branched chain-amino acid transaminase (Thairu et al., 2017). A moderate inhibition of gene expression was observed but the effect varied upon the targeted gene and the aphid species. Before applying this technology to inhibit expression of *Eph* in aphids, additional experiments are required to address dsRNA_Eph_ stability in the environment and efficacy when aerosolized on aphids. However, at this point, it is tempting to make a parallel with the new strategies that are developed to curtail viral human diseases, and in particular Human immunodeficiency virus infection. Indeed, among the therapies to inhibit Human immunodeficiency virus cell entry, a simultaneous knock down of the CCR5 co-receptor by small RNA hairpins together with the expression of an antiviral fusion inhibitor peptide is under a clinical trial (Wolstein et al., 2014; Hutter et al., 2015).

The results presented in this paper pave the way toward a better comprehension of the molecular mechanisms governing poleroviruses transmission by aphids. Expression and localization of the Eph in aphid species differing in their ability to transmit poleroviruses together with the identification of Eph viral ligands and cellular partners need to be addressed in the future.

## Author contributions

VB, BM, MM, and SB: designed the experiments; MM, BM, SB, MR, SM, and NB: performed the experiments; VB, BM, MM, and SB wrote the paper.

### Conflict of interest statement

The authors declare that the research was conducted in the absence of any commercial or financial relationships that could be construed as a potential conflict of interest. The reviewer TT and handling Editor declared their shared affiliation.

## References

[B1] Alford S. Watson-Hurthig A. Scott N. Carette A. Lorimer H. Bazowski J.. (2010). Soluble ephrin a1 is necessary for the growth of HeLa and SK-BR3 cells. Cancer Cell Int. 10:41. 10.1186/1475-2867-10-41 20979646 PMC2984395

[B2] Ammar el D. Tsai C. W. Whitfield A. E. Redinbaugh M. G. Hogenhout S. A. (2009). Cellular and molecular aspects of rhabdovirus interactions with insect and plant hosts. Annu. Rev. Entomol. 54, 447–468. 10.1146/annurev.ento.54.110807.090454 18793103

[B3] Barker H. Waterhouse P. M. (1999). The development of resistance to luteoviruses mediated by host genes and pathogen-derived transgenes, in The Luteoviridae, eds Smith H. G. Barker H. (Wallingford, CT: CAB International), 169–210.

[B4] Blanc S. Drucker M. Uzest M. (2014). Localizing viruses in their insect vectors. Annu. Rev. Phytopathol. 52, 403–425. 10.1146/annurev-phyto-102313-045920 24996011

[B5] Bonaparte M. I. Dimitrov A. S. Bossart K. N. Crameri G. Mungall B. A. Bishop K. A.. (2005). Ephrin-B2 ligand is a functional receptor for Hendra virus and Nipah virus. Proc. Natl. Acad. Sci. U.S.A. 102, 10652–10657. 10.1073/pnas.0504887102 15998730 PMC1169237

[B6] Bouvaine S. Boonham N. Douglas A. E. (2011). Interactions between a luteovirus and the GroEL chaperonin protein of the symbiotic bacterium *Buchnera aphidicola* of aphids. J. Gen. Virol. 92(Pt. 6), 1467–1474. 10.1099/vir.0.029355-0 21346031

[B7] Brault V. Bergdoll M. Mutterer J. Prasad V. Pfeffer S. Erdinger M.. (2003). Effects of point mutations in the major capsid protein of beet western yellows virus on capsid formation, virus accumulation, and aphid transmission. J. Virol. 77, 3247–3256. 10.1128/JVI.77.5.3247-3256.2003 12584348 PMC149785

[B8] Brault V. Herrbach E. Reinbold C. (2007). Electron microscopy studies on luteovirid transmission by aphids. Micron 38, 302–312. 10.1016/j.micron.2006.04.005 16750376

[B9] Brault V. Mutterer J. Scheidecker D. Simonis M. T. Herrbach E. Richards K.. (2000). Effects of point mutations in the readthrough domain of the beet western yellows virus minor capsid protein on virus accumulation in planta and on transmission by aphids. J. Virol. 74, 1140–1148. 10.1128/JVI.74.3.1140-1148.2000 10627524 PMC111448

[B10] Brault V. Perigon S. Reinbold C. Erdinger M. Scheidecker D. Herrbach E.. (2005). The polerovirus minor capsid protein determines vector specificity and intestinal tropism in the aphid. J. Virol. 79, 9685–9693. 10.1128/JVI.79.15.9685-9693.2005 16014930 PMC1181584

[B11] Brault V. van den Heuvel J. F. Verbeek M. Ziegler-Graff V. Reutenauer A. Herrbach E.. (1995). Aphid transmission of beet western yellows luteovirus requires the minor capsid read-through protein P74. EMBO J. 14, 650–659. 7882968 10.1002/j.1460-2075.1995.tb07043.x PMC398128

[B12] Bruyère A. Brault V. Ziegler-Graff V. Simonis M. T. Van den Heuvel J. F. Richards K.. (1997). Effects of mutations in the beet western yellows virus readthrough protein on its expression and packaging and on virus accumulation, symptoms, and aphid transmission. Virology 230, 323–334. 10.1006/viro.1997.8476 9143288

[B13] Burrows M. E. Caillaud M. C. Smith D. M. Benson E. C. Gildow F. E. Gray S. M. (2006). Genetic regulation of polerovirus and luteovirus transmission in the Aphid *Schizaphis graminum*. Phytopathology 96, 828–837. 10.1094/PHYTO-96-0828 18943747

[B14] Burrows M. E. Caillaud M. C. Smith D. M. Gray S. M. (2007). Biometrical genetic analysis of luteovirus transmission in the aphid *Schizaphis graminum*. Heredity 98, 106–113. 10.1038/sj.hdy.6800909 17021612

[B15] Chakraborty S. Veettil M. V. Bottero V. Chandran B. (2012). Kaposi's sarcoma-associated herpesvirus interacts with EphrinA2 receptor to amplify signaling essential for productive infection. Proc. Natl. Acad. Sci. U.S.A. 109, E1163–E1172. 10.1073/pnas.1119592109 22509030 PMC3358903

[B16] Chay C. A. Gunasinge U. B. Dinesh-Kumar S. P. Miller W. A. Gray S. M. (1996). Aphid transmission and systemic plant infection determinants of barley yellow dwarf luteovirus-PAV are contained in the coat protein readthrough domain and 17-kDa protein, respectively. Virology 219, 57–65. 10.1006/viro.1996.0222 8623554

[B17] Cilia M. Tamborindeguy C. Fish T. Howe K. Thannhauser T. W. Gray S. (2011). Genetics coupled to quantitative intact proteomics links heritable aphid and endosymbiont protein expression to circulative polerovirus transmission. J. Virol. 85, 2148–2166. 10.1128/JVI.01504-10 21159868 PMC3067806

[B18] Cossart P. Helenius A. (2014). Endocytosis of viruses and bacteria. Cold Spring Harb. Perspect. Biol. 6:a016972. 10.1101/cshperspect.a016972 25085912 PMC4107984

[B19] Dedryver C. A. Le Ralec A. Fabre F. (2010). The conflicting relationships between aphids and men: a review of aphid damage and control strategies. C. R. Biol. 333, 539–553. 10.1016/j.crvi.2010.03.009 20541165

[B20] Dedryver C. A. Riault G. Tanguy S. Le Gallic J. F. Trottet M. Jacquot E. (2005). Intra-specific variation and inheritance of BYDV-PAV transmission in the aphid *Sitobion avenae*. Eur. J. Plant Pathol. 111, 341–354. 10.1007/s10658-004-4890-1

[B21] Dogimont C. Slama S. Martin J. Lecoq H. Pitrat M. (1996). Sources of resistance to cucurbit aphid-borne yellows luteovirus in a melon germ plasm collection. Plant Dis. 80, 1379–1382. 10.1094/PD-80-1379

[B22] Doumayrou J. Sheber M. Bonning B. C. Miller W. A. (2016). Role of pea enation mosaic virus coat protein in the host plant and aphid vector. Viruses 8:312. 10.3390/v8110312 27869713 PMC5127026

[B23] Dreyer F. Graichen F. Jung C. (2001). A major quantitative trait locus for resistance to *Turnip yellows virus* (TuYV, syn. beet western yellows virus, BWYV) in rapeseed. Plant Breed. 120, 457–462. 10.1046/j.1439-0523.2001.00646.x

[B24] Drucker M. Then C. (2015). Transmission activation in non-circulative virus transmission: a general concept? Curr. Opin. Virol. 15, 63–68. 10.1016/j.coviro.2015.08.006 26318641

[B25] Filichkin S. A. Brumfield S. Filichkin T. P. Young M. J. (1997). *In vitro* interactions of the aphid endosymbiotic SymL chaperonin with barley yellow dwarf virus. J. Virol. 71, 569–577. 8985385 10.1128/jvi.71.1.569-577.1997 PMC191086

[B26] Gardner J. P. Durso R. J. Arrigale R. R. Donovan G. P. Maddon P. J. Dragic T.. (2003). L-SIGN (CD 209L) is a liver-specific capture receptor for hepatitis C virus. Proc. Natl. Acad. Sci. U.S.A. 100, 4498–4503. 10.1073/pnas.0831128100 12676990 PMC153584

[B27] Genander M. Frisen J. (2010). Ephrins and Eph receptors in stem cells and cancer. Curr. Opin. Cell Biol. 22, 611–616. 10.1016/j.ceb.2010.08.005 20810264

[B28] Gildow F. (1999). Luteovirus transmission mechanisms regulating vector specificity, in The Luteoviridae, eds Smith H. G. Barker H. (Oxon: CAB International), 88–111.

[B29] Gildow F. E. Reavy B. Mayo M. A. Duncan G. H. Woodford J. A. Lamb J. W.. (2000). Aphid acquisition and cellular transport of potato leafroll virus-like particles lacking P5 readthrough protein. Phytopathology 90, 1153–1161. 10.1094/PHYTO.2000.90.10.1153 18944480

[B30] Gray S. Cilia M. Ghanim M. (2014). Circulative, “nonpropagative” virus transmission: an orchestra of virus-, insect-, and plant-derived instruments. Adv. Virus Res. 89, 141–199. 10.1016/B978-0-12-800172-1.00004-5 24751196

[B31] Gray S. Gildow F. E. (2003). Luteovirus-aphid interactions. Annu. Rev. Phytopathol. 41, 539–566. 10.1146/annurev.phyto.41.012203.105815 12730400

[B32] Grove J. Marsh M. (2011). The cell biology of receptor-mediated virus entry. J. Cell Biol. 195, 1071–1082. 10.1083/jcb.201108131 22123832 PMC3246895

[B33] Guilley H. Wipf-Scheibel C. Richards K. Lecoq H. Jonard G. (1994). Nucleotide sequence of cucurbit aphid-borne yellows luteovirus. Virology 202, 1012–1017. 10.1006/viro.1994.1429 8030201

[B34] Hahn A. S. Desrosiers R. C. (2013). Rhesus monkey rhadinovirus uses eph family receptors for entry into B cells and endothelial cells but not fibroblasts. PLoS Pathog. 9:e1003360 10.1371/journal.ppat.1003360 23696734 PMC3656109

[B35] Hahn A. S. Kaufmann J. K. Wies E. Naschberger E. Panteleev-Ivlev J. Schmidt K.. (2012). The ephrin receptor tyrosine kinase A2 is a cellular receptor for Kaposi's sarcoma-associated herpesvirus. Nat. Med. 18, 961–966. 10.1038/nm.2805 22635007 PMC3645317

[B36] Harrewijn P. (1983). The effect of cultural measures on behaviour and population development of potato aphids and transmission of viruses. Mededelingen Faculteit Landbouwwetenschappen Rijksuniversiteit Gent 48, 791–798.

[B37] Herrbach E. (1999). Effect of luteovirus infection on vector, in The Luteoviridae, eds Smith H. G. Barker H. (Wallingford: CABI), 123–125.

[B38] Himanen J. P. Saha N. Nikolov D. B. (2007). Cell-cell signaling via Eph receptors and ephrins. Curr. Opin. Cell Biol. 19, 534–542. 10.1016/j.ceb.2007.08.004 17928214 PMC3327877

[B39] Hipper C. Monsion B. Bortolamiol-Becet D. Ziegler-Graff V. Brault V. (2014). Formation of virions is strictly required for turnip yellows virus long-distance movement in plants. J. Gen. Virol. 95(Pt. 2), 496–505. 10.1099/vir.0.058867-0 24214396

[B40] Hogenhout S. A. Ammar el D. Whitfield A. E. Redinbaugh M. G. (2008). Insect vector interactions with persistently transmitted viruses. Annu. Rev. Phytopathol. 46, 327–359. 10.1146/annurev.phyto.022508.092135 18680428

[B41] Hutter G. Bodor J. Ledger S. Boyd M. Millington M. Tsie M.. (2015). CCR5 targeted cell therapy for HIV and prevention of viral escape. Viruses 7, 4186–4203. 10.3390/v7082816 26225991 PMC4576177

[B42] Kania A. Klein R. (2016). Mechanisms of ephrin-Eph signalling in development, physiology and disease. Nat. Rev. Mol. Cell Biol. 17, 240–256. 10.1038/nrm.2015.16 26790531

[B43] Kaplan I. B. Lee L. Ripoll D. R. Palukaitis P. Gildow F. Gray S. M. (2007). Point mutations in the potato leafroll virus major capsid protein alter virion stability and aphid transmission. J. Gen. Virol. 88(Pt. 6), 1821–1830. 10.1099/vir.0.82837-0 17485544

[B44] Kaushansky A. Douglass A. N. Arang N. Vigdorovich V. Dambrauskas N. Kain H. S.. (2015). Malaria parasites target the hepatocyte receptor EphA2 for successful host infection. Science 350, 1089–1092. 10.1126/science.aad3318 26612952 PMC4783171

[B45] Lecoq H. Bourdin D. Wipf-Scheibel C. Bon M. Lot H. Lemaire O. (1992). A new yellowing disease of cucurbits caused by a luteovirus, cucurbit aphid-borne yellows virus. Plant Pathol. 41, 749–761. 10.1111/j.1365-3059.1992.tb02559.x

[B46] Leiser R. M. Ziegler-Graff V. Reutenauer A. Herrbach E. Lemaire O. Guilley H.. (1992). Agroinfection as an alternative to insects for infecting plants with beet western yellows luteovirus. Proc. Natl. Acad. Sci. U.S.A. 89, 9136–9140. 10.1073/pnas.89.19.9136 1409615 PMC50080

[B47] Li C. Cox-Foster D. Gray S. M. Gildow F. (2001). Vector specificity of barley yellow dwarf virus (BYDV) transmission: identification of potential cellular receptors binding BYDV-MAV in the aphid, *Sitobion avenae*. Virology 286, 125–133. 10.1006/viro.2001.0929 11448166

[B48] Linz L. B. Liu S. Chougule N. P. Bonning B. C. (2015). *In vitro* evidence supports membrane alanyl aminopeptidase N as a receptor for a plant virus in the pea aphid vector. J. Virol. 89, 11203–11212. 10.1128/JVI.01479-15 26311872 PMC4645670

[B49] Liu S. Sivakumar S. Sparks W. O. Miller W. A. Bonning B. C. (2010). A peptide that binds the pea aphid gut impedes entry of *Pea enation mosaic virus* into the aphid hemocoel. Virology 401, 107–116. 10.1016/j.virol.2010.02.009 20223498

[B50] Liu S. Sivakumar S. Wang Z. Bonning B. C. Miller W. A. (2009). The readthrough domain of pea enation mosaic virus coat protein is not essential for virus stability in the hemolymph of the pea aphid. Arch. Virol. 154, 469–479. 10.1007/s00705-009-0327-7 19240978

[B51] Lupberger J. Zeisel M. B. Xiao F. Thumann C. Fofana I. Zona L.. (2011). EGFR and EphA2 are host factors for hepatitis C virus entry and possible targets for antiviral therapy. Nat. Med. 17, 589–595. 10.1038/nm.2341 21516087 PMC3938446

[B52] Marjomaki V. Pietiainen V. Matilainen H. Upla P. Ivaska J. Nissinen L.. (2002). Internalization of echovirus 1 in caveolae. J. Virol. 76, 1856–1865. 10.1128/JVI.76.4.1856-1865.2002 11799180 PMC135881

[B53] Marston D. J. Dickinson S. Nobes C. D. (2003). Rac-dependent trans-endocytosis of ephrinBs regulates Eph-ephrin contact repulsion. Nat. Cell Biol. 5, 879–888. 10.1038/ncb1044 12973357

[B54] Mercer J. Schelhaas M. Helenius A. (2010). Virus entry by endocytosis. Annu. Rev. Biochem. 79, 803–833. 10.1146/annurev-biochem-060208-104626 20196649

[B55] Mulot M. Boissinot S. Monsion B. Rastegar M. Clavijo G. Halter D.. (2016). Comparative analysis of RNAi-based methods to down-regulate expression of two genes expressed at different levels in *Myzus persicae*. Viruses 8:316. 10.3390/v8110316 27869783 PMC5127030

[B56] Ng J. C. Falk B. W. (2006). Virus-vector interactions mediating nonpersistent and semipersistent transmission of plant viruses. Annu. Rev. Phytopathol. 44, 183–212. 10.1146/annurev.phyto.44.070505.143325 16602948

[B57] Papura D. Jacquot E. Dedryver C. A. Luche S. Riault G. Bossis M.. (2002). Two-dimensional electrophoresis of proteins discriminates aphid clones of *Sitobion avenae* differing in BYDV-PAV transmission. Arch. Virol. 147, 1881–1898. 10.1007/s00705-002-0859-6 12376751

[B58] Pasquale E. B. (2005). Eph receptor signalling casts a wide net on cell behaviour. Nat. Rev. Mol. Cell Biol. 6, 462–475. 10.1038/nrm1662 15928710

[B59] Pelkmans L. Kartenbeck J. Helenius A. (2001). Caveolar endocytosis of simian virus 40 reveals a new two-step vesicular-transport pathway to the ER. Nat. Cell Biol. 3, 473–483. 10.1038/35074539 11331875

[B60] Perez White B. E. Getsios S. (2014). Eph receptor and ephrin function in breast, gut, and skin epithelia. Cell Adh. Migr. 8, 327–338. 10.4161/19336918.2014.970012 25482622 PMC4594571

[B61] Peter K. A. Liang D. Palukaitis P. Gray S. M. (2008). Small deletions in the potato leafroll virus readthrough protein affect particle morphology, aphid transmission, virus movement and accumulation. J. Gen. Virol. 89(Pt. 8), 2037–2045. 10.1099/vir.0.83625-0 18632976

[B62] Pitulescu M. E. Adams R. H. (2010). Eph/ephrin molecules–a hub for signaling and endocytosis. Genes Dev. 24, 2480–2492. 10.1101/gad.1973910 21078817 PMC2975924

[B63] Reinbold C. Gildow F. E. Herrbach E. Ziegler-Graff V. Goncalves M. C. van Den Heuvel J. F.. (2001). Studies on the role of the minor capsid protein in transport of Beet western yellows virus through *Myzus persicae*. J. Gen. Virol. 82(Pt. 8), 1995–2007. 10.1099/0022-1317-82-8-1995 11458007

[B64] Rejman J. Bragonzi A. Conese M. (2005). Role of clathrin- and caveolae-mediated endocytosis in gene transfer mediated by lipo- and polyplexes. Mol. Ther. 12, 468–474. 10.1016/j.ymthe.2005.03.038 15963763

[B65] Seddas P. Boissinot S. Strub J. M. Van Dorsselaer A. Van Regenmortel M. H. Pattus F. (2004). Rack-1, GAPDH3, and actin: proteins of *Myzus persicae* potentially involved in the transcytosis of beet western yellows virus particles in the aphid. Virology 325, 399–412. 10.1016/j.virol.2004.05.014 15246278

[B66] Stapleton D. Balan I. Pawson T. Sicheri F. (1999). The crystal structure of an Eph receptor SAM domain reveals a mechanism for modular dimerization. Nat. Struct. Biol. 6, 44–49. 10.1038/4917 9886291

[B67] Stevens M. Freeman B. Liu H. Y. Herrbach E. Lemaire O. (2005). Beet poleroviruses: close friends or distant relatives? Mol. Plant Pathol. 6, 1–9. 10.1111/j.1364-3703.2004.00258.x 20565633

[B68] Subbarayal P. Karunakaran K. Winkler A. C. Rother M. Gonzalez E. Meyer T. F.. (2015). EphrinA2 receptor (EphA2) is an invasion and intracellular signaling receptor for *Chlamydia trachomatis*. PLoS Pathog. 11:e1004846. 10.1371/journal.ppat.1004846 25906164 PMC4408118

[B69] Sylvester E. S. (1967). Retention of inoculativity in the transmission of *Pea enation mosaic virus* by pea aphids as associated with virus isolates, aphid reproduction and excretion. Virology 32, 524–531. 10.1016/0042-6822(67)90304-2 6028944

[B70] Thairu M. W. Skidmore I. H. Bansal R. Novakova E. Hansen T. E. Li-Byarlay H.. (2017). Efficacy of RNA interference knockdown using aerosolized short interfering RNAs bound to nanoparticles in three diverse aphid species. Insect Mol. Biol. 26, 356–368. 10.1111/imb.12301 28314050

[B71] Thomsen P. Roepstorff K. Stahlhut M. van Deurs B. (2002). Caveolae are highly immobile plasma membrane microdomains, which are not involved in constitutive endocytic trafficking. Mol. Biol. Cell 13, 238–250. 10.1091/mbc.01-06-0317 11809836 PMC65085

[B72] Tjallingii W. F. (1985). Membrane potentials as an indication for plant cell penetration by aphid stylets. Entomol. Exp. Appl. 38, 187–193. 10.1111/j.1570-7458.1985.tb03517.x

[B73] Torrance L. (1992). Analysis of epitopes on potato leafroll virus capsid protein. Virology 191, 485–489. 10.1016/0042-6822(92)90216-C 1384231

[B74] Van den Heuvel F. Peters D. (1990). Transmission of potato leafroll virus in relation to the honeydew excretion of Myzus persicae. Ann. Appl. Biol. 116, 493–502. 10.1111/j.1744-7348.1990.tb06632.x

[B75] Van den Heuvel J. F. Boerma T. M. Peters D. (1991). Transmission of potato leafroll virus from plants and artificial diets by *Myzus persicae*. Phytopathology 81, 150–154. 10.1094/Phyto-81-150

[B76] van den Heuvel J. F. Bruyere A. Hogenhout S. A. Ziegler-Graff V. Brault V. Verbeek M.. (1997). The N-terminal region of the luteovirus readthrough domain determines virus binding to Buchnera GroEL and is essential for virus persistence in the aphid. J. Virol. 71, 7258–7265. 9311800 10.1128/jvi.71.10.7258-7265.1997 PMC192067

[B77] van den Heuvel J. F. Verbeek M. Van der Wilk F. (1994). Endosymbiotic bacteria associated with circulative transmission of potato leafroll virus by *Myzus persicae*. J. Gen. Virol. 75(Pt. 10), 2559–2565. 10.1099/0022-1317-75-10-2559 7931143

[B78] Veidt I. Bouzoubaa S. E. Leiser R. M. Ziegler-Graff V. Guilley H. Richards K.. (1992). Synthesis of full-length transcripts of beet western yellows virus RNA: messenger properties and biological activity in protoplasts. Virology 186, 192–200. 10.1016/0042-6822(92)90073-X 1727597

[B79] von Hahn T. Rice C. M. (2008). Hepatitis C virus entry. J. Biol. Chem. 283, 3689–3693. 10.1074/jbc.R700024200 17881349

[B80] Walkey D. G. A. Pink D. A. C. (1990). Studies on resistance to beet western yellows virus in lettuce (*Lactuca sativa*) and the occurrence of field sources of the virus. Plant Pathol. 39, 141–155. 10.1111/j.1365-3059.1990.tb02485.x

[B81] Wang X. Zhou G. (2003). Identification of a protein associated with circulative transmission of barley yellow dwarf virus from cereal aphids, *Schizaphis graminum* and *Sitobion avenae*. Chin. Sci. Bull. 48, 2083–2087. 10.1360/03wc0153

[B82] Wang Y. Gosselin Grenet A. S. Castelli I. Cermenati G. Ravallec M. Fiandra L.. (2013). Densovirus crosses the insect midgut by transcytosis and disturbs the epithelial barrier function. J. Virol. 87, 12380–12391. 10.1128/JVI.01396-13 24027326 PMC3807927

[B83] Whitfield A. E. Falk B. W. Rotenberg D. (2015). Insect vector-mediated transmission of plant viruses. Virology 479–480, 278–289. 10.1016/j.virol.2015.03.026 25824478

[B84] Wolstein O. Boyd M. Millington M. Impey H. Boyer J. Howe A.. (2014). Preclinical safety and efficacy of an anti-HIV-1 lentiviral vector containing a short hairpin RNA to CCR5 and the C46 fusion inhibitor. Mol. Ther. Methods Clin. Dev. 1:11. 10.1038/mtm.2013.11 26015947 PMC4365823

[B85] Yang X. Thannhauser T. W. Burrows M. Cox-Foster D. Gildow F. E. Gray S. M. (2008). Coupling genetics and proteomics to identify aphid proteins associated with vector-specific transmission of polerovirus (Luteoviridae). J. Virol. 82, 291–299. 10.1128/JVI.01736-07 17959668 PMC2224398

[B86] Zimmer M. Palmer A. Kohler J. Klein R. (2003). EphB-ephrinB bi-directional endocytosis terminates adhesion allowing contact mediated repulsion. Nat. Cell Biol. 5, 869–878. 10.1038/ncb1045 12973358

