# Supplementary material for: Transmission of Turnip yellows virus by Myzus persicae Is Reduced by Feeding Aphids on Double-Stranded RNA Targeting the Ephrin Receptor Protein
Source: Front Microbiol. 2018 Mar 13;9:457. doi: 10.3389/fmicb.2018.00457 (PMC5859162; doi:10.3389/fmicb.2018.00457)
Supplement: Supplementary file 2 [file Presentation1.PPTX]

## Slide 1
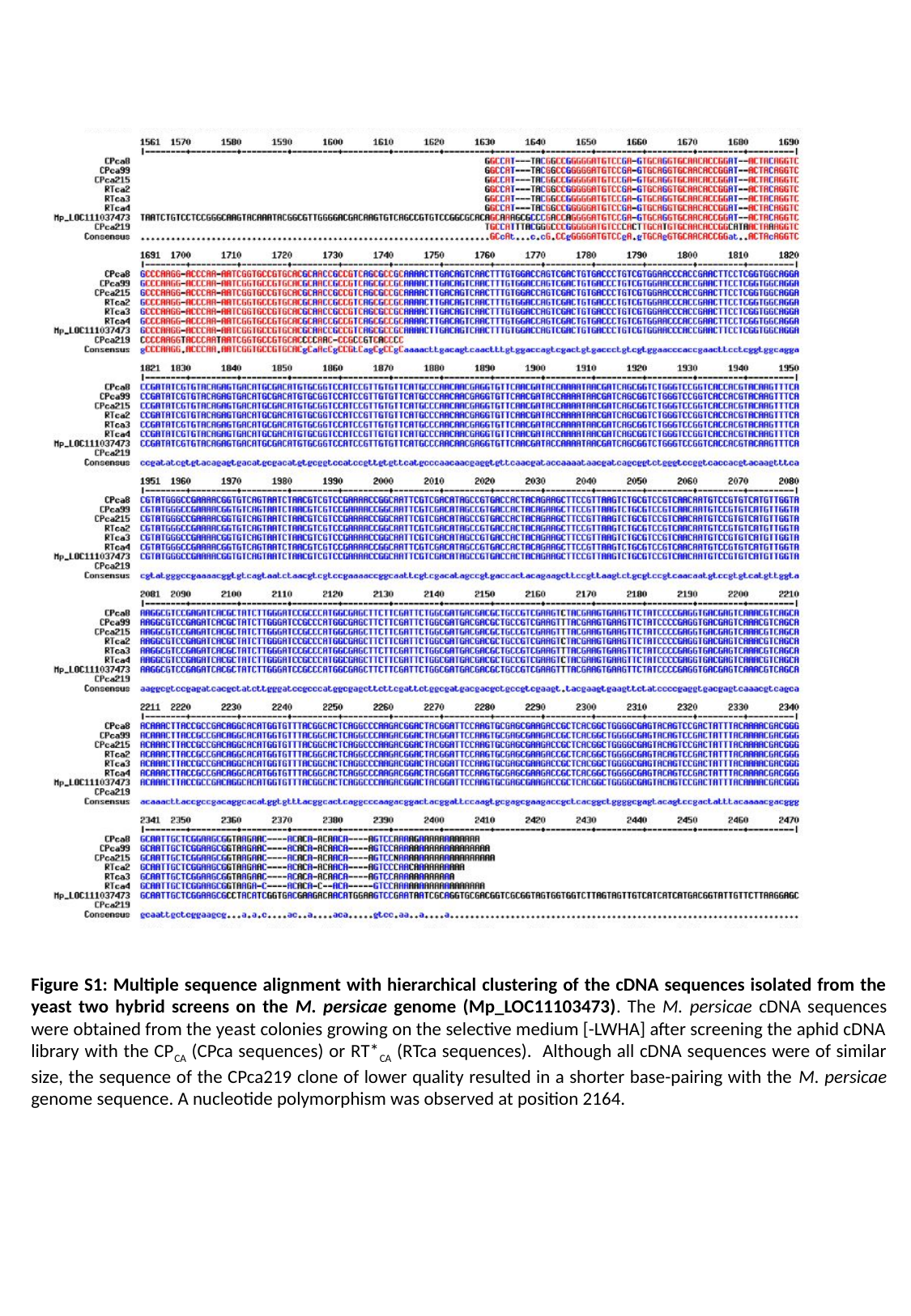

Figure S1: Multiple sequence alignment with hierarchical clustering of the cDNA sequences isolated from the yeast two hybrid screens on the M. persicae genome (Mp_LOC11103473). The M. persicae cDNA sequences were obtained from the yeast colonies growing on the selective medium [-LWHA] after screening the aphid cDNA library with the CPCA (CPca sequences) or RT*CA (RTca sequences). Although all cDNA sequences were of similar size, the sequence of the CPca219 clone of lower quality resulted in a shorter base-pairing with the M. persicae genome sequence. A nucleotide polymorphism was observed at position 2164.
