# Supplementary material for: Transmission of Turnip yellows virus by Myzus persicae Is Reduced by Feeding Aphids on Double-Stranded RNA Targeting the Ephrin Receptor Protein
Source: Front Microbiol. 2018 Mar 13;9:457. doi: 10.3389/fmicb.2018.00457 (PMC5859162; doi:10.3389/fmicb.2018.00457)
Supplement: Supplementary file 3 [file Presentation2.PPTX]

## Slide 1
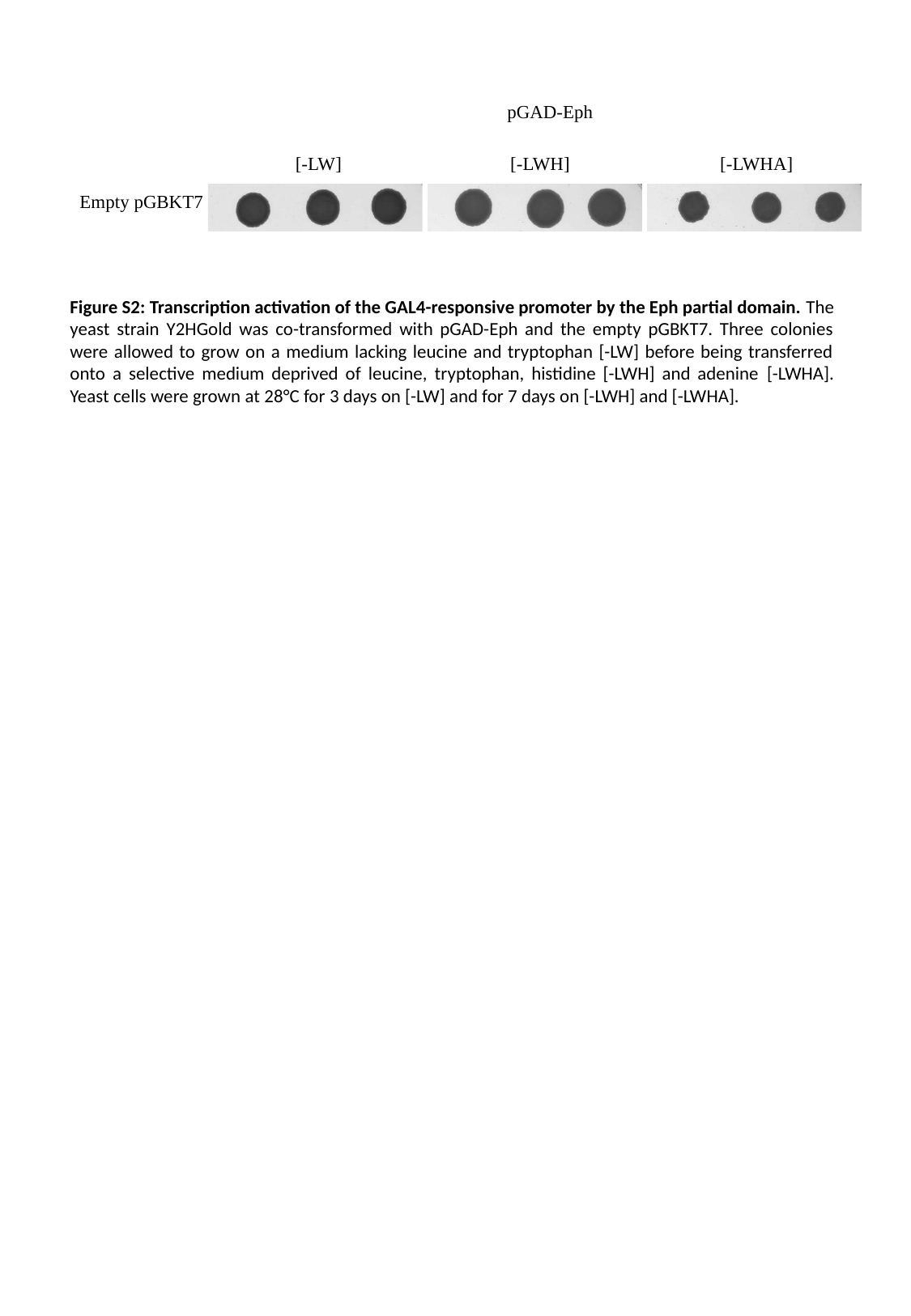

pGAD-Eph
[-LW]
[-LWH]
[-LWHA]
Empty pGBKT7
Figure S2: Transcription activation of the GAL4-responsive promoter by the Eph partial domain. The yeast strain Y2HGold was co-transformed with pGAD-Eph and the empty pGBKT7. Three colonies were allowed to grow on a medium lacking leucine and tryptophan [-LW] before being transferred onto a selective medium deprived of leucine, tryptophan, histidine [-LWH] and adenine [-LWHA]. Yeast cells were grown at 28°C for 3 days on [-LW] and for 7 days on [-LWH] and [-LWHA].
