# Supplementary material for: Transmission of Turnip yellows virus by Myzus persicae Is Reduced by Feeding Aphids on Double-Stranded RNA Targeting the Ephrin Receptor Protein
Source: Front Microbiol. 2018 Mar 13;9:457. doi: 10.3389/fmicb.2018.00457 (PMC5859162; doi:10.3389/fmicb.2018.00457)
Supplement: Supplementary file 4 [file Presentation3.PPTX]

## Slide 1
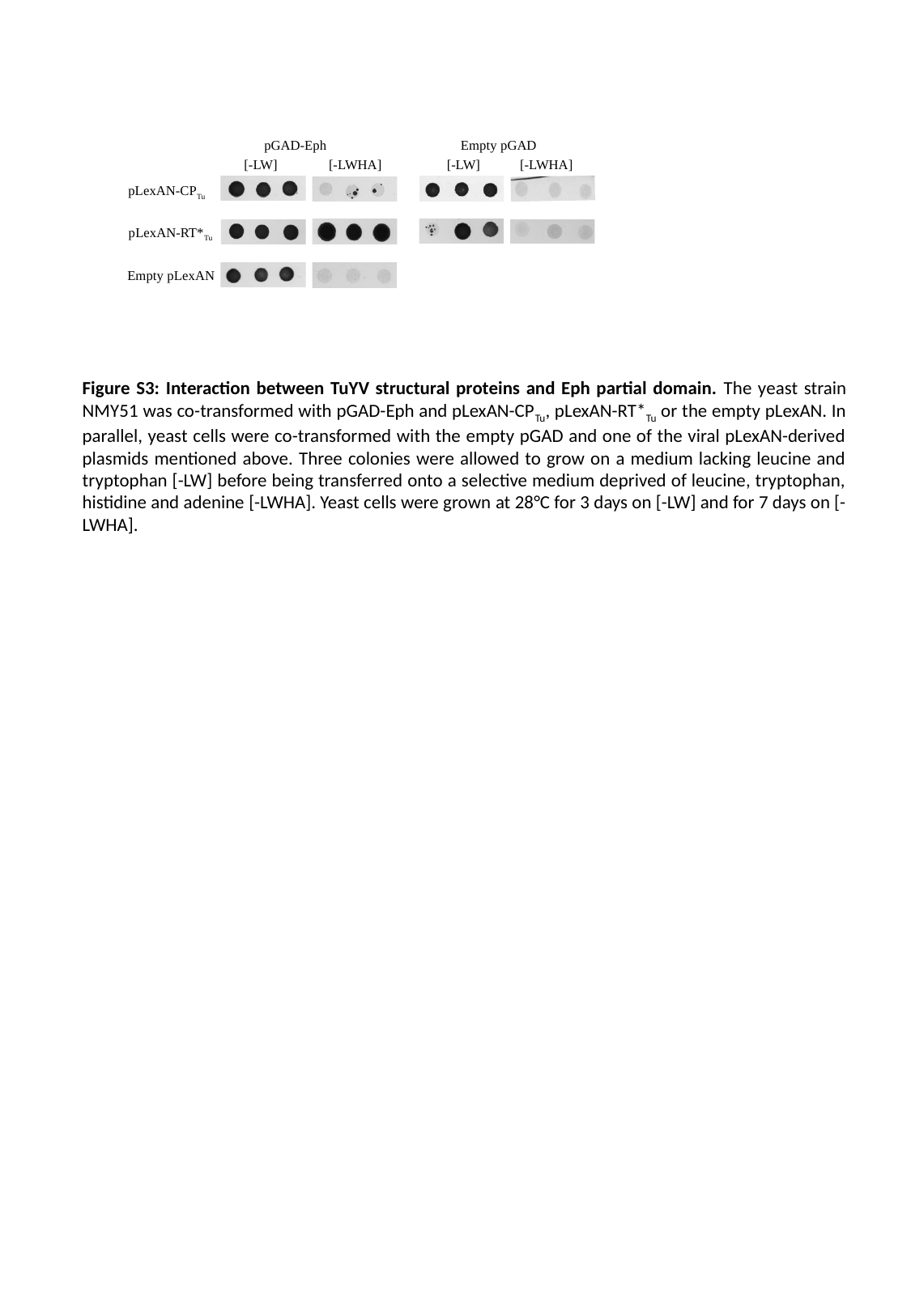

pGAD-Eph
Empty pGAD
[-LW]
[-LWHA]
[-LW]
[-LWHA]
pLexAN-CPTu
pLexAN-RT*Tu
Empty pLexAN
Figure S3: Interaction between TuYV structural proteins and Eph partial domain. The yeast strain NMY51 was co-transformed with pGAD-Eph and pLexAN-CPTu, pLexAN-RT*Tu or the empty pLexAN. In parallel, yeast cells were co-transformed with the empty pGAD and one of the viral pLexAN-derived plasmids mentioned above. Three colonies were allowed to grow on a medium lacking leucine and tryptophan [-LW] before being transferred onto a selective medium deprived of leucine, tryptophan, histidine and adenine [-LWHA]. Yeast cells were grown at 28°C for 3 days on [-LW] and for 7 days on [-LWHA].
