# Supplementary material for: Transmission of Turnip yellows virus by Myzus persicae Is Reduced by Feeding Aphids on Double-Stranded RNA Targeting the Ephrin Receptor Protein
Source: Front Microbiol. 2018 Mar 13;9:457. doi: 10.3389/fmicb.2018.00457 (PMC5859162; doi:10.3389/fmicb.2018.00457)
Supplement: Supplementary file 5 [file Presentation4.PPTX]

## Slide 1
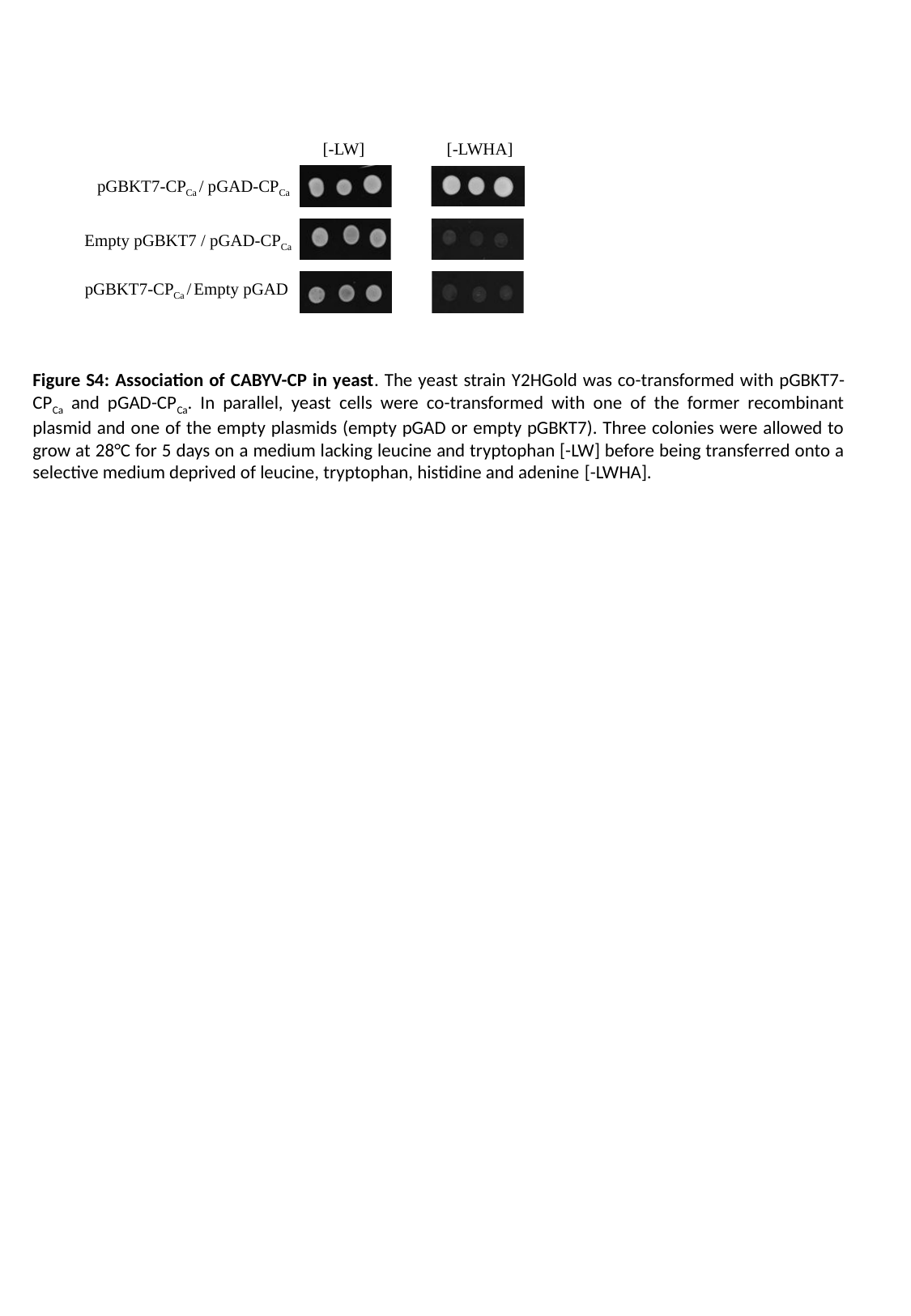

[-LW]
[-LWHA]
pGBKT7-CPCa / pGAD-CPCa
Empty pGBKT7 / pGAD-CPCa
pGBKT7-CPCa / Empty pGAD
Figure S4: Association of CABYV-CP in yeast. The yeast strain Y2HGold was co-transformed with pGBKT7-CPCa and pGAD-CPCa. In parallel, yeast cells were co-transformed with one of the former recombinant plasmid and one of the empty plasmids (empty pGAD or empty pGBKT7). Three colonies were allowed to grow at 28°C for 5 days on a medium lacking leucine and tryptophan [-LW] before being transferred onto a selective medium deprived of leucine, tryptophan, histidine and adenine [-LWHA].
