# Supplementary material for: Transmission of Turnip yellows virus by Myzus persicae Is Reduced by Feeding Aphids on Double-Stranded RNA Targeting the Ephrin Receptor Protein
Source: Front Microbiol. 2018 Mar 13;9:457. doi: 10.3389/fmicb.2018.00457 (PMC5859162; doi:10.3389/fmicb.2018.00457)
Supplement: Supplementary file 6 [file Presentation5.PPTX]

## Slide 1
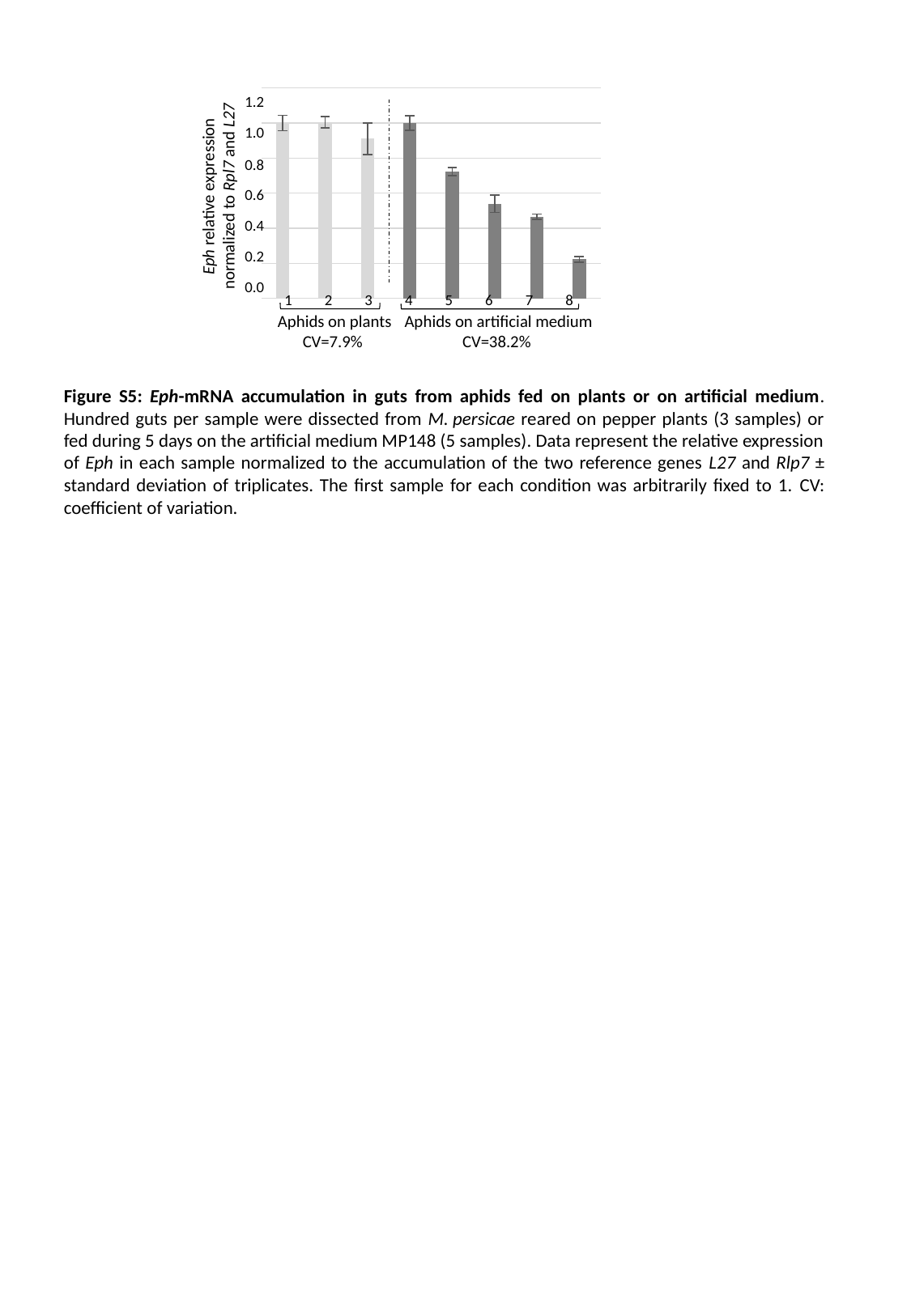

### Chart
| Category | |
|---|---|1.2
1.0
0.8
Eph relative expression normalized to Rpl7 and L27
0.6
0.4
0.2
0.0
1
2
3
4
5
6
7
8
Aphids on plants
CV=7.9%
Aphids on artificial medium
CV=38.2%
Figure S5: Eph-mRNA accumulation in guts from aphids fed on plants or on artificial medium. Hundred guts per sample were dissected from M. persicae reared on pepper plants (3 samples) or fed during 5 days on the artificial medium MP148 (5 samples). Data represent the relative expression of Eph in each sample normalized to the accumulation of the two reference genes L27 and Rlp7 ± standard deviation of triplicates. The first sample for each condition was arbitrarily fixed to 1. CV: coefficient of variation.
