# Supplementary material for: Transmission of Turnip yellows virus by Myzus persicae Is Reduced by Feeding Aphids on Double-Stranded RNA Targeting the Ephrin Receptor Protein
Source: Front Microbiol. 2018 Mar 13;9:457. doi: 10.3389/fmicb.2018.00457 (PMC5859162; doi:10.3389/fmicb.2018.00457)
Supplement: Supplementary file 7 [file Presentation6.PPTX]

## Slide 1
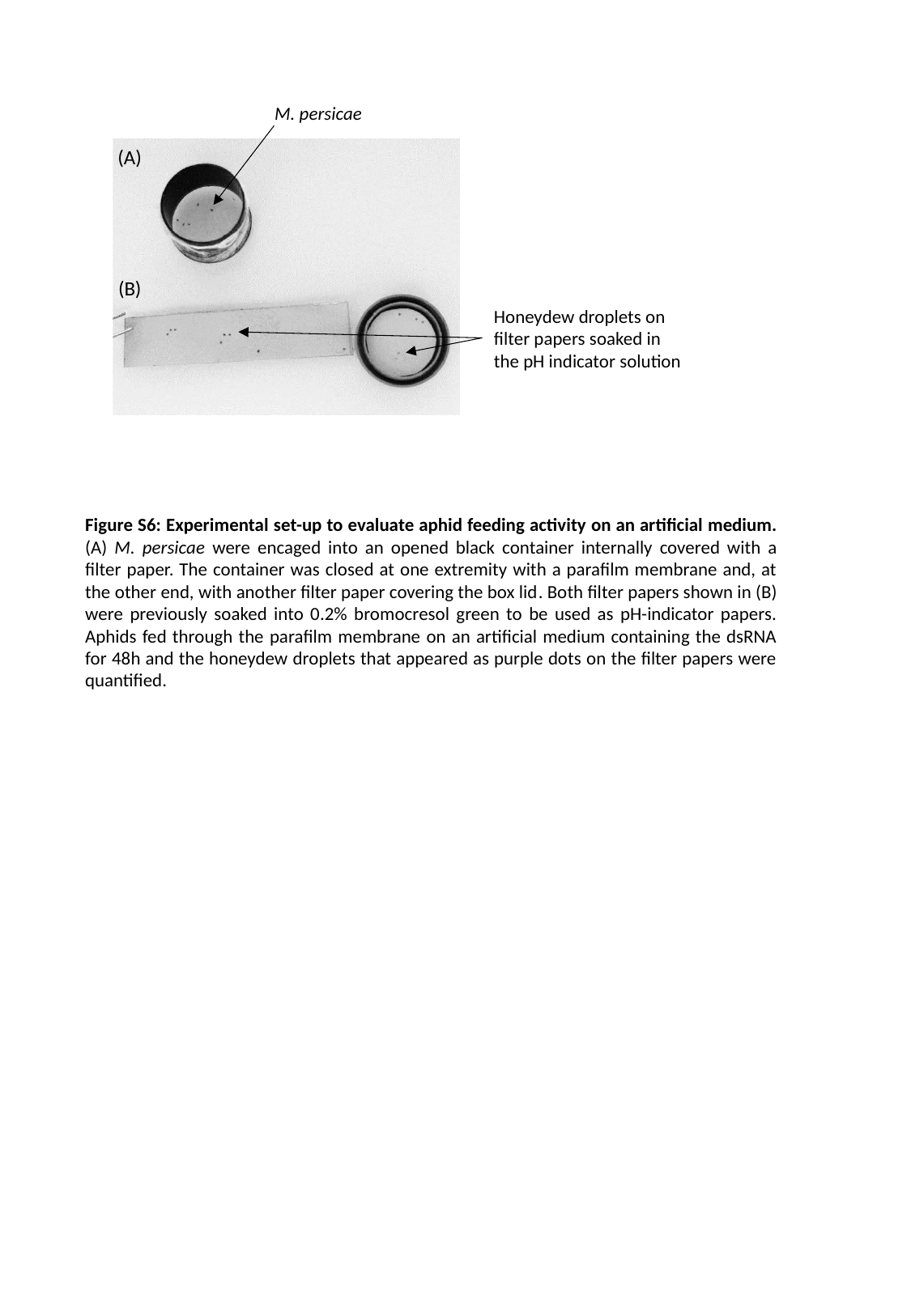

M. persicae
(A)
(B)
Honeydew droplets on
filter papers soaked in the pH indicator solution
Figure S6: Experimental set-up to evaluate aphid feeding activity on an artificial medium. (A) M. persicae were encaged into an opened black container internally covered with a filter paper. The container was closed at one extremity with a parafilm membrane and, at the other end, with another filter paper covering the box lid. Both filter papers shown in (B) were previously soaked into 0.2% bromocresol green to be used as pH-indicator papers. Aphids fed through the parafilm membrane on an artificial medium containing the dsRNA for 48h and the honeydew droplets that appeared as purple dots on the filter papers were quantified.
