# Supplementary material for: Transmission of Turnip yellows virus by Myzus persicae Is Reduced by Feeding Aphids on Double-Stranded RNA Targeting the Ephrin Receptor Protein
Source: Front Microbiol. 2018 Mar 13;9:457. doi: 10.3389/fmicb.2018.00457 (PMC5859162; doi:10.3389/fmicb.2018.00457)
Supplement: Supplementary file 8 [file Presentation7.PPTX]

## Slide 1
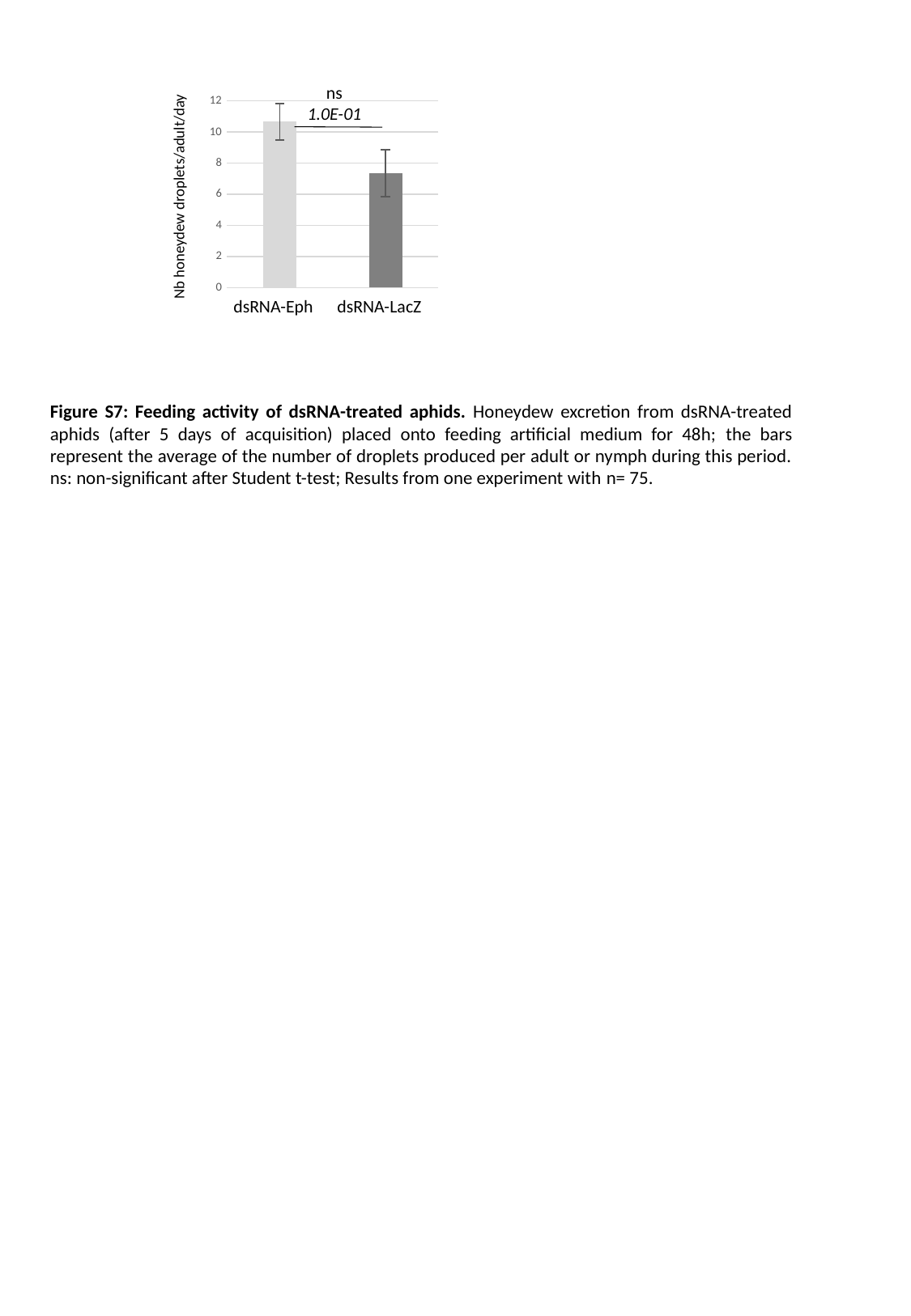

### Chart
| Category | |
|---|---|ns
1.0E-01
Nb honeydew droplets/adult/day
dsRNA-LacZ
dsRNA-Eph
Figure S7: Feeding activity of dsRNA-treated aphids. Honeydew excretion from dsRNA-treated aphids (after 5 days of acquisition) placed onto feeding artificial medium for 48h; the bars represent the average of the number of droplets produced per adult or nymph during this period. ns: non-significant after Student t-test; Results from one experiment with n= 75.
